# Informed Weighted Non-Negative Matrix Factorization Using *αβ*-Divergence Applied to Source Apportionment

**DOI:** 10.3390/e21030253

**Published:** 2019-03-06

**Authors:** Gilles Delmaire, Mahmoud Omidvar, Matthieu Puigt, Frédéric Ledoux, Abdelhakim Limem, Gilles Roussel, Dominique Courcot

**Affiliations:** 1 Laboratoire LISIC–EA 4491, Université du Littoral Côte d’Opale, F-62228 Calais, France; 2 Laboratoire UCEIV–EA 4492, Université du Littoral Côte d’Opale, SFR CONDORCET FR CNRS 3417, F-59140 Dunkerque, France

**Keywords:** non-negative matrix factorization, informed NMF, robust cost function, source apportionment, air pollution

## Abstract

In this paper, we propose informed weighted non-negative matrix factorization (NMF) methods using an αβ-divergence cost function. The available information comes from the exact knowledge/boundedness of some components of the factorization—which are used to structure the NMF parameterization—together with the row sum-to-one property of one matrix factor. In this contribution, we extend our previous work which partly involved some of these aspects to αβ-divergence cost functions. We derive new update rules which are extendthe previous ones and take into account the available information. Experiments conducted for several operating conditions on realistic simulated mixtures of particulate matter sources show the relevance of these approaches. Results from a real dataset campaign are also presented and validated with expert knowledge.

## 1. Introduction

Source apportionment consists of estimating the particulate matter (PM) sources present in the ambient air together with their relative concentrations. A source is fully characterized by a profile which gathers the *m* chemical species’ proportions (expressed in ng/μg) that constitute it. Usually, several, say *n*, PM samples are collected using an automated sampler, then characterized to asses the chemical composition. Each of them can be written as a mixture of *p* profiles, with different concentrations (expressed in ng/m${}^{3}$). Mathematically, if we respectively denote by *X*, *G*, and *F* as the non-negative n×m data matrix, n×p contribution matrix, and p×m profile matrix, the collected data reads

(1) X≈G·F.

While being known under the name of (blind) source separation in the signal/image processing community, Equation (1) is called the receptor model in the chemistry community. In practice, the latter should satisfy the following properties [1]:

1. The entries of *G* and *F* are non-negative (one cannot assume a negative mass in *G* nor a negative proportion of chemical species in *F*).
2. The product G·F must fit the data matrix *X*.
3. When one entry of the product ${\left(G\;\cdot \;F\right)}_{ij}$ does not fit the entry $x_{ij}$, we should then check
  (2) $$x_{ij}⪰{\left(G\;\cdot \;F\right)}_{ij},$$
  i.e., the estimated mass of a chemical species in a sample should not be above the corresponding measured one.

As a consequence, estimating the unknown matrices *G* and *F* is mainly performed using positive matrix factorization (PMF) [2] and, in particular, using its popular version from the US Environmental Protection Agency.

Independently from the PMF investigations done by the chemistry community, Equation (1) has been massively considered by the signal/image processing and the machine learning communities which processed it with non-negative matrix factorization (NMF) techniques [3].

The general idea behind NMF is to minimize a discrepancy measure between *X* and the estimated product G·F. Such a problem has been extensively studied in the past years. Historically—apart from pioneering work [4]—most methods are based on an alternating optimization of the factor matrices. NMF has been massively investigated because of the more interpretable results it provides when compared with methods without sign constraints. NMF was successfully applied to many fields, e.g., hyperspectral unmixing [5,6], astrophysics [7,8], fluorescence spectroscopy for agro-food analysis [9], audio signals [10], or environmental data processing [11].

It should be noticed that NMF is flexible and can take into consideration additional assumptions to provide a better estimation of the NMF factors. In the literature, assumptions such as sparseness [12,13], fixed row and/or column sums [13,14], structure in the matrix factors [15,16], or orthogonality constraints [17] were investigated.

Solving Equation (1) can be performed by appropriately choosing a discrepancy measure between *X* and GF. When this measure is the Frobenius norm of their difference, the possible presence of a few outliers may corrupt the NMF enhancement. As a consequence, robust NMF methods were proposed to deal with a predefined number of outliers. While some of them decompose the data matrix into the sum of a low-rank and a sparse matrix—where the latter contains the outlying component [18]—most ones consider some modified cost functions as dissimilarity measures which gave rise to flexible and robust algorithms, e.g., Bregman-NMF [19], α-NMF [20], β-NMF [21,22], αβ-NMF [23], Correntropy-NMF [24], Huber-NMF [25] (it should be noticed that the Huber cost function has also been considered for robust PMF [26]).

Lastly, it should be noticed that in receptor models, each data point $x_{ij}$ is provided with an uncertainty measure $\sigma_{ij}$ and PMF actually solves a weighted optimization problem [4,26]. Weighted extensions of NMF have been also considered, e.g., to enhance the factorization [27] or to deal with missing entries [28,29]. However, it is known than both the PMF [30] and the standard NMF techniques face some convergence issues (however, the convergence of NMF is guaranteed under some separability assumptions [3] which are not satisfied in practice in the considered application and which are thus out of the scope of this paper) [3].

As a consequence, we investigated the enhancement provided by informed NMF. In Ref. [31], the use of a Gaussian plume model enables us to assess the presence or absence of some punctual sources, depending on wind measures, and source and sensor locations which allowed us to fix some entries of *G* to zero. In the absence of a punctual source, such an information should be dropped. In Ref. [32], an informed NMF-based weighted criterion takes into consideration the known values of some terms of *F* (Informed NMF has also been proposed in [33] where the known entries are seen as a penalization term in the NMF optimization problem) in order to improve the separation. For that purpose, we introduced a specific parameterization for NMF methods using a Frobenius norm. This approach should be considered as a flexible NMF counterpart of [34] in between blind source separation—where no information on *F* is provided—and regression, where *F* is fully known. While it was shown in practice to be less sensitive than blind NMF to convergence issues, this method can still be affected by outliers which are present in many receptor modeling problems.

In this paper, we thus extend our previous work [32] by (i) investigating and discussing several αβ-divergence expressions, (ii) exploring different data normalization procedures combined with set values (as profiles are chemical species proportions, the rows of *F* are normalized), and (iii) adding minimum and maximum bounds to some of the unknown values of *F*. The methods we propose in this paper have been partially introduced in [35,36], in the framework of the β-divergence only. We generalize here [35,36] to the αβ-divergence and we provide a detailed study of their performance, shown on both realistic simulations and real data campaign.

The remainder of the paper is structured as follows. We recall some properties of the αβ-divergence in Section 2. Section 3 introduces our proposed NMF parameterization—which puts on light the special structure of the profile matrix in the NMF algorithm—while Section 4 is dedicated to the problem formulation. We introduce our proposed methods in Section 5 that we test in Section 6. Lastly, we conclude about the proposed work in Section 7. Appendix A introduces update rules for an alternative informed αβ-NMF method.

## 2. Robust Cost Functions

### 2.1. Introduction to Modified Cost Functions

Chemical data often face some particular measures whose characteristics substantially differ from those which are commonly observed. From a signal processing point of view, such data may be considered as outliers which may degrade the performance of classical algorithms using the Frobenius norm in their cost function. Such an issue is often addressed in the field of robustness where the challenge is to design new algorithms which take into account the above corrupted data.

Apart from the low-rank plus sparse decomposition [18], robust NMF algorithms using modified cost functions were investigated. Indeed, these robust functions provide less penalization to large entries of the residual matrix, which is defined as

(3) R≜X−G·F.

Among them, the Huber cost function accounts for the differentiable connection between the $\ell_{2}$ and $\ell_{1}$ norms, according to the residual value with respect to an adaptive cutoff parameter. Another popular modified cost function stands for the correntropy measure [24] which accounts for a bounded and non-convex discrepancy measure.

**Figure 1:**
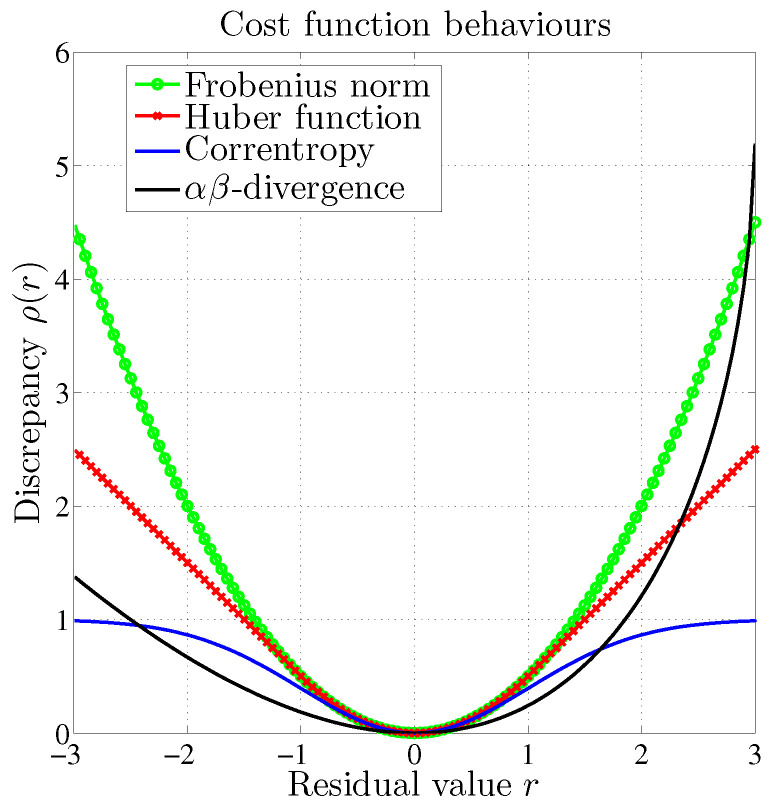
Behavior of several dissimilarity measures with respect to the residual value.

In contrast with the above measures, the αβ-divergence is not a norm as it is not symmetrical. Figure 1 shows an example of the behavior of such functions which are penalizing the values of the residual in different ways. As mentioned earlier, the αβ-divergence is the only cost function to present a possible asymmetric behavior around the null residual value. Hopke [1] highlighted the need for methods dedicated to chemical source apportionment which enforce a positive residual value. This situation fits well with the configuration described in Figure 1.

### 2.2. αβ-Divergence

The αβ-divergence (For special values of α,β, the reader is invited to consult [23]) is a parametric discrepancy measure which may be used to evaluate the gap between two scalar quantities *p* and *q*, i.e., ∀(α,β,α+β)≠0,

(4) $$D^{\alpha ,\beta }\left(p||q\right)=\frac{-1}{\alpha \beta }\left(p^{\alpha }q^{\beta }-\frac{\alpha }{\alpha +\beta }p^{\alpha +\beta }-\frac{\beta }{\alpha +\beta }q^{\alpha +\beta }\right).$$

Special values of the parameters lead to very famous divergence measures [23], such as α-divergences or β-divergences [21]. These divergences are different from classical norms in the sense that they check some common properties—e.g., non-negativity—while others such as symmetry, scalability and triangular inequality are not satisfied.

Cichocki et al. [23] study the influence of the parameters α and β on the robustness of the estimated data (they also establish general connections between the general αβ-divergence and the scaled $\frac{\alpha }{\alpha +\beta }$-order α-divergence with an α+β zoom of its arguments). To this aim, they express the sensitivity to outliers by computing the differentiation with respect to an unknown parameter here replaced for simplicity with an entry of *F*, namely $F_{rj}$, i.e.,

(5) $$\frac{\partial D^{\alpha ,\beta }\left(X∥\hat{X}\right)}{\partial F_{rj}}=-\sum_{i}\frac{\partial {\hat{X}}_{i,j}}{\partial F_{rj}}\underset{\mathrm{weight}}{\underset{︸}{{\left({\hat{X}}_{ij}\right)}^{\alpha +\beta -1}}}\;\underset{\alpha -\mathrm{zoom}}{\underset{︸}{\ln_{1-\alpha }\left(X_{ij}/{\hat{X}}_{ij}\right)}},$$

where

(6) $${\hat{X}}_{ij}≜\sum_{r}G_{ir}F_{rj}≜{\left(G\;\cdot \;F\right)}_{ij},$$

and

(7) $$\ln_{1-\alpha }\left(z\right)=\left\{\begin{array}{ccc} \frac{z^{\alpha }-1}{\alpha }, & \mathrm{if} & \alpha \neq 0,\\ \ln(z), & \mathrm{if} & \alpha =0. \end{array}\right.$$

Considering Equation (6), the expression $\frac{\partial {\hat{X}}_{i,j}}{\partial F_{rj}}=G_{ir}$ may be replaced in Equation (5), leading to

(8) $$\frac{\partial D^{\alpha ,\beta }\left(X∥\hat{X}\right)}{\partial F_{rj}}=-\sum_{i}G_{ir}\underset{\alpha \beta \;\mathrm{weight}}{\underset{︸}{{\left({\hat{X}}_{ij}\right)}^{\alpha +\beta -1}}}\;\underset{\alpha -\mathrm{zoom}}{\underset{︸}{\ln_{1-\alpha }\left(X_{ij}/{\hat{X}}_{ij}\right)}}$$

For the sake of comparison, sensitivity equations of M-estimators [37] are usually designed for the weighted Frobenius cost function (corresponding to α=1 and β=1 in a αβ-divergence), i.e.,

(9) $$\frac{\partial D^{1,1}\left(X∥\hat{X}\right)}{\partial F_{rj}}=-\sum_{i}G_{ir}\underset{\mathrm{weight}}{\underset{︸}{W_{ij}}}\;\underset{\mathrm{Residual}\;\mathrm{entry}}{\underset{︸}{{\left(X-G\;\cdot \;F\right)}_{ij}}}$$

where $W_{ij}$ accounts for the general entry of the weight matrix. This weight is usually viewed as a confidence index into the corresponding data. As a consequence, a large residual together with a large weight leads to large modifications in the estimates. In the frame of Equation (8), the weight entry reads

(10) $$W_{ij}=\frac{{\left({\hat{X}}_{ij}\right)}^{\alpha +\beta -1}\;ln_{1-\alpha }\left(X_{ij}/{\hat{X}}_{ij}\right)}{R_{ij}}=\underset{\alpha \beta \;\mathrm{weight}}{\underset{︸}{{\left({\hat{X}}_{ij}\right)}^{\alpha +\beta -1}}}\underset{\alpha -\mathrm{zoom}\;\mathrm{weight}}{\underset{︸}{\frac{1}{\alpha }\frac{{\left(X_{ij}\right)}^{\alpha }-{\left({\hat{X}}_{ij}\right)}^{\alpha }}{\left(X_{ij}-{\hat{X}}_{ij}\right)}}}.$$

**Figure 2:**
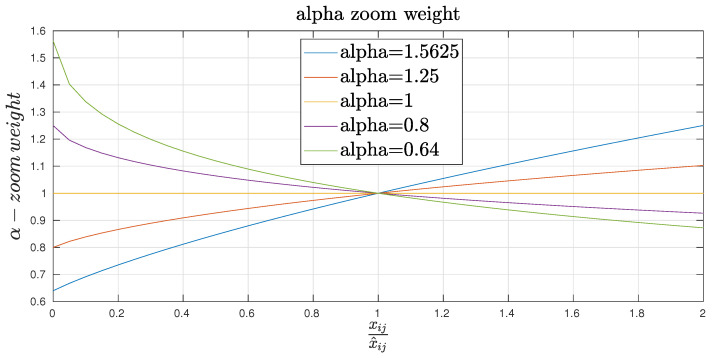
The α-zoom weight.

Figure 2 describes the α-zoom weight as a function of the ratio $\frac{X_{ij}}{{\hat{X}}_{ij}}$ for different values of α. It turns out that α<1 provides small weight to large values of the ratio $\frac{X_{ij}}{{\hat{X}}_{ij}}$. In other words, this situation does not induce big changes in the estimates. Outliers such as $X_{ij}\geq {\hat{X}}_{ij}$ will be allowed in this context.

Equation (8) combines two effects, namely an α-zoom and an αβ weight effect. When α>1, the emphasis of the α-zoom is put on larger values of the ratio $\frac{X_{ij}}{{\hat{X}}_{ij}}$ while the emphasis is put on smaller values of this ratio when α<1. These properties are recalled in Table 1. The αβ weight effect in ${\left({\hat{X}}_{ij}\right)}^{\alpha +\beta -1}$ is expressed as a function of α+β in Table 2.

**Figure 3:**
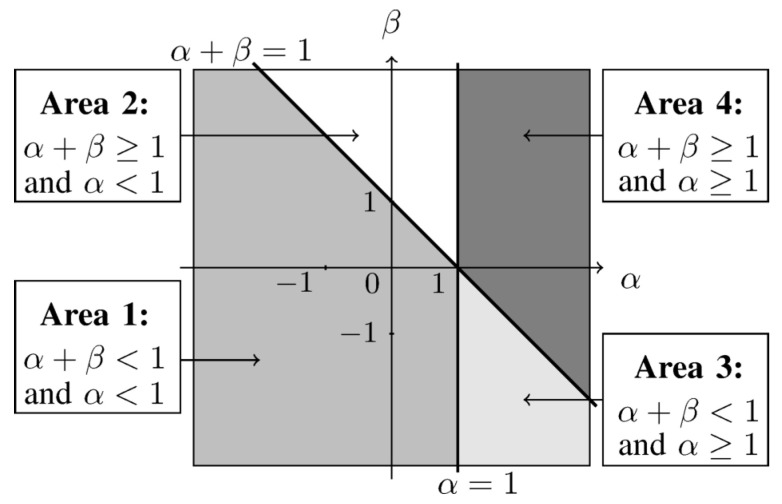
Different areas as a function of α and β.

To summarize, α can be used to control the influence of large or small ratios in the estimator through the α-zoom, while β provides some control on the weighting of the ratios depending on the demand to better fit to larger or smaller values of the model [23]. Gathering these properties, the space of values (α,β) may be partitioned in several areas as described in Figure 3.

Each zone allows a certain kind of outliers. Areas 1 and 2 allow outliers of the form $X_{ij}>{\hat{X}}_{ij}$ for large and small amplitudes of ${\hat{X}}_{ij}$, respectively. Areas 3 and 4 accept outliers such as $X_{ij}<{\hat{X}}_{ij}$ for large and small amplitudes of ${\hat{X}}_{ij}$, respectively. Areas 1 and 3 favor a better fit to small values of *X* while areas 2 and 4 favor a better fit to large ones. As a consequence, for our considered application, we propose to favor a best fit for major species with respect to minor species. This leads to considering the case α+β>1. Secondly, if the estimation does not fit the data, we prefer keeping situations where $X_{ij}>{\hat{X}}_{ij}$ holds, as explained in Section 1 and in [1]. This fact results in the choice α<1. These two conditions give rise to an area of interest which is area 2 and which is kept along the article (for convexity reasons in NMF [23], area 2 should be delimited to β<1).

### 2.3. Existing NMF Methods with Parametric Divergences

NMF methods are formulated as the global minimization of a cost function under the non-negativity of both factors *G* and *F*. Aside from pioneering work [4], NMF is classically performed through an iterative procedure which alternatively minimizes—for a fixed *F* (respectively *G*)—a discrepancy between *X* and G·F. Multiplicative update rules were firstly proposed in [38] for the Frobenius norm and the Kullback–Leibler divergence. While being easy to implement, multiplicative algorithms only ensure that the cost function does not increase within iterations, which is not sufficient for getting a limit point. The study of NMF convergence through the Karush–Kuhn Tucker (KKT) conditions was explored by Lin [39]; stationarity is only a necessary condition of a local minimum. Moreover, some limit points which are not stationary may exist, especially if some components of *F* and *G* are initialized to zero.

Moreover, most algorithms are sensitive to the initialization and to the presence of outliers. Parametric divergences may reduce the influence of this last drawback by an appropriate choice of the hyperparameters.

Cichocki et al. [20] proposed multiplicative update rules with α-divergence. The developed rules were based on the majorization-minimization (MM) strategy [40] but they may also be obtained in a heuristic way by using the KKT conditions or partial derivatives of the cost function as well.

Févotte and Idier [21] proposed to use the β-divergence as a cost function and derived different kinds of rules according to three different strategies involving the heuristic approach, the majorization-minimization strategy [40] and a new one called majorization-equalization. This last strategy provides a larger step size and a faster convergence. Hennequin et al. stated that the β-divergence could be viewed as a special case of Bregman divergence [41], thus leading us to apply Bregman divergence theorems to β-divergence. Cichocki et al. [23] proposed NMF based on generalized αβ-divergences in the framework of majorization-minimization (MM).

Extending the work in [27,42] from the one hand and in [23] from the other hand, we introduced in [35] a weighted β-NMF (β-WNMF) defined for β∈[0;1]. It is straightforward to extend it to a Weighted αβ-NMF which amounts to minimizing a weighted αβ-divergence,

(11) $$\min_{G⪰0,F⪰0}D_{W}^{\alpha ,\beta }\left(X∥G\;\cdot \;F\right)≜\min_{G⪰0,F⪰0}\sum_{i,j}W_{ij}\;D^{\alpha ,\beta }\left(x_{ij}∥{\left(G\;\cdot \;F\right)}_{ij}\right),$$

and yields

(12) $$F^{k+1}=F^{k}\circ N_{F}^{\alpha ,\beta }\left(G^{k},F^{k}\right),\;G^{k+1}=G^{k}\circ N_{G}^{\alpha ,\beta }\left(G^{k},F^{k}\right),$$

where

(13) $$N_{F}^{\alpha ,\beta }\left(G,F\right)≜{\left[\frac{G^{T}\;\cdot \;\left(W\circ X^{\alpha }\circ {\left(G\;\cdot \;F\right)}^{\beta -1}\right)}{G^{T}\;\cdot \;\left(W\circ {\left(G\;\cdot \;F\right)}^{\alpha +\beta -1}\right)}\right]}^{\frac{1}{\alpha }},$$

(14) $$N_{G}^{\alpha ,\beta }\left(G,F\right)≜{\left[\frac{\left(W\circ X^{\alpha }\circ {\left(G\;\cdot \;F\right)}^{\beta -1}\right)\;\cdot \;F^{T}}{\left(W\circ {\left(G\;\cdot \;F\right)}^{\alpha +\beta -1}\right)\;\cdot \;F^{T}}\right]}^{\frac{1}{\alpha }},$$

and X∘Y and $\frac{X}{Y}$ respectively denote the componentwise product and division between two matrices. *W* is a weight matrix used to model the uncertainties $\sigma_{ij}$ associated to the data samples $x_{ij}$, and whose general element $w_{ij}$ is set to $w_{ij}≜\sigma_{ij}^{-(\alpha +\beta )}$. This approach encompasses several other methods, especially αβ-NMF [23] if $W=1_{nm}$, i.e., for any *i* and *j*, $w_{ij}=1$, and β-NMF [21] if additionally α=1.

Apart from multiplicative updates, NMF based on alternating direction method of multipliers (ADMM) were recently proposed [43] for their ability to perform distributed computations for large scale data and in particular, Sun and Févotte introduced an approach based on the β-divergence [22] while Zhu and Honeine [24] proposed a correntropy-based approach for large deviations. Such fast approaches are not required for the considered chemical application where the global computation time is not an issue.

## 3. Constraint Parameterization

In this paper, we assume the values of some components of the profile matrix *F* to be provided or bounded by experts. We thus propose a formalism which takes into account this knowledge. It extends our previous parameterization [32] which only considered equality constraints.

Let $\Omega^{E}$ and $\Omega^{I}$ be two p×m binary matrices which inform the presence/absence of equality and inequality constraints on each element $f_{ij}$ of the matrix *F*, respectively, i.e.,

(15) $$\omega_{ij}^{E}=\left\{\begin{array}{cc} 1 & \mathrm{if}\;f_{ij}\;\mathrm{is}\;\mathrm{known},\\ 0 & \mathrm{otherwise}, \end{array}\right.\omega_{ij}^{I}=\left\{\begin{array}{cc} 1 & \mathrm{if}\;f_{ij}\;\mathrm{is}\;\mathrm{bounded},\\ 0 & \mathrm{otherwise}. \end{array}\right.$$

We then define the p×m binary matrices ${\bar{\Omega }}^{E}$ and ${\bar{\Omega }}^{I}$ as ${\bar{\Omega }}^{E}≜1_{pm}-\Omega^{E}$ and ${\bar{\Omega }}^{I}≜1_{pm}-\Omega^{I}$, where $1_{pm}$ is the p×m matrix of ones. By construction, we obtain

(16) $$\Omega^{E}\circ \Omega^{I}=0,\;\Omega^{I}⪯{\bar{\Omega }}^{E}.$$

We denote by $\Phi^{E}$ the p×m sparse matrix of set values, i.e.,

(17) $$\Phi^{E}≜F\circ \Omega^{E}.$$

Please note that $\varphi_{ij}^{E}$—the (i,j)-th element of $\Phi^{E}$—is equal to zero when $\omega_{ij}^{E}=0$. We can easily prove that

(18) $$\Phi^{E}\circ \Omega^{E}=\Phi^{E},\;\Phi^{E}\circ {\bar{\Omega }}^{E}=0.$$

Similarly, we define $\Phi^{I+}$ and $\Phi^{I-}$ the p×m sparse matrices of upper and lower bounds (equality constraints could be considered as inequalities, with the same upper and lower bounds. However, in some preliminary tests, we found our proposed approaches to outperform those using bound constraints only), respectively, i.e.,

(19) $$\Phi^{I-}⪯F\circ \Omega^{I}⪯\Phi^{I+}.$$

Let $f_{i}$ and $\varphi_{i}^{E}$ be the *i*-th column of *F* and $\Phi^{E}$, respectively. A column $f_{i}$ may be expressed as

(20) $$\;f_{i}=\varphi_{i}^{E}+\Gamma_{i}\theta_{i},$$

where $\theta_{i}$ and $\Gamma_{i}$ are respectively the $(p-l_{i})\times 1$ vector of free parameters and the $p\times (p-l_{i})$ orthonormal basis of free parameters [32]. From Equation (20), we define $\Delta f_{i}$ as

(21) $$\Delta f_{i}≜f_{i}-\varphi_{i}^{E}=\Gamma_{i}\theta_{i},$$

and ΔF as the matrix gathering each column $\Delta f_{i}$, i.e.,

(22) $$\Delta F≜F-\Phi^{E}.$$

Following the stages in [32]—which combine Equations (17), (18) and (22)—we obtain the matrix form of Equation (20):

(23) $$F=\Omega^{E}\circ \Phi^{E}+{\bar{\Omega }}^{E}\circ \Delta F.$$

This expression of *F* puts on light its specific structure, as *F* is expressed as the sum of its set and free parts. Moreover, combining Equations (16) and (23) leads to

(24) $$F=\Omega^{E}\circ \Phi^{E}+{\bar{\Omega }}^{E}\circ {\bar{\Omega }}^{I}\circ \Delta F+{\bar{\Omega }}^{E}\circ \Omega^{I}\circ \Delta F,$$

which shows that the free part of *F* may be decomposed as a bounded part and an unconstrained one.

## 4. General Problem Formulation

The proposed informed NMF methods consist of estimating the matrices *G* and *F* in order to get an approximate factorization (1) under the above constraints, i.e.,

(25) $$\min_{G⪰0,F⪰0}D_{W}^{\alpha ,\beta }\left(X∥G\;\cdot \;F\right)s.t.\left\{\begin{array}{c} F\circ \Omega^{E}=\Phi^{E},\\ \Phi^{I-}⪯F\circ \Omega^{I}⪯\Phi^{I+},\\ F\;\cdot \;1_{mm}=1_{pm}, \end{array}\right.$$

where the weighted divergence $D_{W}^{\alpha ,\beta }\left(\;\cdot \;∥\;\cdot \;\right)$ is defined in Equation (11). The first constraint ensures that some predefined components of *F* are set while the second one forces the selected components to be bound-constrained. The last condition enforces each row of *F* to be normalized, i.e., $\sum_{j=1}^{m}f_{ij}=1$, ∀i=1…p (Please note that the normalization met in remote sensing [44]—where the sum of each row of *F* is equal to one—is not similar, except in a noiseless case in the framework of exact factorization. Moreover, the normalization also differs from the one met in mobile sensor calibration [13] as the normalization is approximately satisfied in the latter).

The main challenge in the Equation (25) consists of finding solutions which are satisfying all the above constraints. The first constraint leads to consider the parametrization (23) that we used in [32]. By substituting the parametrization (23), Equation (25) becomes a constrained NMF with respect to *G* and ΔF, i.e.,

(26) $$\begin{array}{c} \min_{G⪰0,\Delta F⪰0}D_{W}^{\alpha ,\beta }\left(X||G\;\cdot \;\left(\Omega^{E}\circ \Phi^{E}\right)+G\;\cdot \;\left({\bar{\Omega }}^{E}\circ \Delta F\right)\right)\\ s.t.\left\{\begin{array}{c} \Phi^{I-}⪯\Delta F\circ \Omega^{I}⪯\Phi^{I+},\\ \Delta F\;\cdot \;1_{mm}=1_{pm}-\Phi^{E}\;\cdot \;1_{mm}. \end{array}\right. \end{array}$$

The last condition is derived from the last one in Equation (25) combined with Equation (22).

In the case of bound constraints only, no dedicated parameterization exists, but projective methods have been developed [45]. The row sum-to-one constraint has been taken under account by using a special parameterization in [14]. However, dealing with all the constraints together at the same time is a difficult task. We thus propose a less elegant, yet efficient strategy which consists of considering them sequentially. By dropping the bound constraint, we obtain the following reduced problem:

(27) $$\begin{array}{c} \min_{G⪰0,\Delta F⪰0}D_{W}^{\alpha ,\beta }\left(X||G\;\cdot \;\left(\Omega^{E}\circ \Phi^{E}\right)+G\;\cdot \;\left({\bar{\Omega }}^{E}\circ \Delta F\right)\right)\\ s.t.\;\Delta F\;\cdot \;1_{mm}=1_{pm}-\Phi^{E}\;\cdot \;1_{mm}. \end{array}$$

As an alternative to the above problem, please note that by combining Equations (1) and (23), we obtain

(28) $$X-G\;\cdot \;\left(\Omega^{E}\circ \Phi^{E}\right)\approx G\;\cdot \;\left({\bar{\Omega }}^{E}\circ \Delta F\right).$$

We can thus derive a slightly different problem, i.e.,

(29) $$\begin{array}{c} \min_{G⪰0,\Delta F⪰0}D_{W}^{\alpha ,\beta }\left(X-G\;\cdot \;\left(\Omega^{E}\circ \Phi^{E}\right)\left|\right|G\;\cdot \;\left({\bar{\Omega }}^{E}\circ \Delta F\right)\right)\\ s.t.\;\Delta F\;\cdot \;1_{mm}=1_{pm}-\Phi^{E}\;\cdot \;1_{mm}, \end{array}$$

which yieldsslightly different update rules. We proposed in [35] some multiplicative update rules to solve Equation (29) in the case of β-divergence only. The extension to the αβ-divergence is derived in Appendix A.

As explained above, instead of looking for the solution of Equation (26) directly, we sequentially consider each additional set of information, i.e., we first estimate ΔF and *F* that we then normalize and project onto the set of admissible solutions (or that we project and then normalize, respectively) within iterations.

## 5. Proposed Informed αβ-NMF Methods

### 5.1. Weighted αβ-NMF with Set Constraints

In this section, we firstly aim to solve Equation (27) without the sum-to-one constraint. The whole strategy follows the majoration-minimization technique [40] and consists of (i) finding a majoring function which is convex with respect to the unknown parameters, and (ii) minimizing this auxiliary function instead of the original one.

**Proposition** **1.** *Update rules for the free part of the profile matrix are*

(30) $$▵F^{k+1}\leftarrow ▵F^{k}\circ {\bar{\Omega }}^{E}\circ N_{F}^{\alpha ,\beta }\left(G^{k},F^{k}\right),$$

*where (denoting λ≜α+β−1), we define*

(31) $$N_{F}^{\alpha ,\beta }\left(G,F\right)≜{\left(\frac{G^{T}\left(W\circ X^{\lambda }\circ {\left(X-G\Phi^{E}\right)}^{1-\beta }\circ {\left(G(▵F)\right)}^{\beta -1}\right)}{G^{T}\left(W\circ X^{\lambda }\circ {\left(X-G\Phi^{E}\right)}^{-\lambda }\circ {\left(G\left(▵F\right)\right)}^{\lambda }\right)}\right)}^{{}^{\frac{1}{\alpha }}}.$$

**Proof.**  We consider a column of the data since the divergence may be split into independent partial divergences. Using the notations defined in Section 3, we hereafter drop the index *i* for the vectors $▵{\underline{f}}_{i}≜\Gamma_{i}{\underline{\theta }}_{i}$, ${\underline{\varphi }}_{i}^{E}$, ${\underline{\theta }}_{i}$, and for the matrix $\Gamma_{i}$. Let *k* be the current iteration index and let us define

(32) U≜GΓ.

Expression (32) together with Equation (21) provide

(33) $$D_{\underline{w}}^{\alpha ,\beta }\left(\underline{x}∥G{\underline{\varphi }}^{E}+G\Delta \underline{f}\right)=D_{\underline{w}}^{\alpha ,\beta }\left(\underline{x}∥G{\underline{\varphi }}^{E}+U\underline{\theta }\right).$$

The weighted αβ-divergence between two corresponding column vectors reads

(34) $$D_{\underline{w}}^{\alpha ,\beta }\left(\underline{x}∥G\underline{f}\right)=\sum_{i}\;w_{i}x_{i}^{\alpha +\beta }\;h^{\alpha ,\beta }\left(\frac{{\left(G{\underline{\varphi }}^{E}\right)}_{i}+\sum_{j}u_{ij}\theta_{j}}{x_{i}}\right),$$

where ∀(α,β,α+β)≠0,

(35) $$h^{\alpha ,\beta }\left(z\right)≜\frac{1}{\alpha \beta }\left[\frac{\alpha }{\alpha +\beta }+\frac{\beta }{\alpha +\beta }z^{\alpha +\beta }-z^{\beta }\right].$$

Provided that $h^{\alpha ,\beta }\left(1\right)=0$ and noticing that $h^{\alpha ,\beta }\left(z\right)$ is convex for z≥0 and β∈[min(1,1−α);max(1,1−α)] [23], Jensen’s inequality may be applied twice, i.e.,

(36) $$h^{\alpha ,\beta }\left(\frac{{\left(G{\underline{\varphi }}^{E}\right)}_{i}+\sum_{j}u_{ij}\theta_{j}}{x_{i}}\right)\;\leq \;\frac{{\left(x-G{\underline{\varphi }}^{E}\right)}_{i}}{x_{i}}\;h^{\alpha ,\beta }\left(\frac{\sum_{j}u_{ij}\theta_{j}}{{\left(x-G{\underline{\varphi }}^{E}\right)}_{i}}\right)$$

and

(37) $$h^{\alpha ,\beta }\left(\frac{\sum_{j}u_{ij}\theta_{j}}{{\left(\underline{x}-G{\underline{\varphi }}^{E}\right)}_{i}}\right)\;\leq \;\sum_{j}\frac{u_{ij}\theta_{j}^{k}}{\sum_{l}u_{il}\theta_{l}^{k}}\;h^{\alpha ,\beta }\left(\frac{\theta_{j}\sum_{l}u_{il}\theta_{l}^{k}}{{\left(x-G{\underline{\varphi }}^{E}\right)}_{i}\theta_{j}^{k}}\right),$$

where the superscript *k* is the current iteration number and $\theta_{j}$ is the *j*-th element of the free parameter vector $\underline{\theta }$ introduced in Equation (20). Equation (34) together with expressions (36) and (37) yield the following auxiliary function:

(38) $$H_{2,w}^{\alpha ,\beta }\left(\theta_{j},\theta_{j}^{k}\right)=\sum_{i}w_{i}\;x_{i}^{\alpha +\beta -1}{\left(\underline{x}-G{\underline{\varphi }}^{E}\right)}_{i}\sum_{j}\frac{u_{ij}\;\theta_{j}^{k}}{\sum_{l}u_{il}\;\theta_{l}^{k}}\;\cdot \;h^{\alpha ,\beta }\left(\frac{\theta_{j}\;\sum_{l}u_{il}\;\theta_{l}^{k}}{{\left(\underline{x}-G{\underline{\varphi }}^{E}\right)}_{i}\;\theta_{j}^{k}}\right).$$

Canceling its gradient $\frac{\partial H_{2,w}^{\alpha ,\beta }\left(\theta_{j},\theta_{j}^{k}\right)}{\partial \theta_{j}}$ leads to the optimum, i.e.,

(39) $${\left(\frac{\theta_{j}}{\theta_{j}^{k}}\right)}^{{}^{\alpha }}=\frac{\sum_{i}w_{i}u_{ij}{\left(\underline{x}-G{\underline{\varphi }}^{E}\right)}_{i}^{1-\beta }x_{i}^{\lambda }{\left(\sum_{l}u_{il}\theta_{l}^{k}\right)}^{\beta -1}}{\sum_{i}w_{i}u_{ij}x_{i}^{\lambda }{\left(\underline{x}-G{\underline{\varphi }}^{E}\right)}_{i}^{-\lambda }{\left(\sum_{l}u_{il}\theta_{l}^{k}\right)}^{\lambda }},$$

which reads in its vector form

(40) $${\left(\frac{\underline{\theta }}{{\underline{\theta }}^{k}}\right)}^{{}^{\alpha }}=\frac{U^{T}\left[\underline{w}\circ {\underline{x}}^{\lambda }\circ {\left(\underline{x}-G{\underline{\varphi }}^{E}\right)}^{1-\beta }\circ {\left(U{\underline{\theta }}^{k}\right)}^{\beta -1}\right]}{U^{T}\left[\underline{w}\circ {\underline{x}}^{\lambda }\circ {\left(\underline{x}-G{\underline{\varphi }}^{E}\right)}^{-\lambda }\circ {\left(U{\underline{\theta }}^{k}\right)}^{\lambda }\right]}.$$

By combining Equation (21) with the above relationship, we derive the expression of one column of the matrix ▵F:

(41) $$\frac{▵{\underline{f}}^{k+1}}{▵{\underline{f}}^{k}}={\left(\frac{\Gamma \;U^{T}\;\left[\underline{w}\circ {\underline{x}}^{\lambda }\circ {\left(\underline{x}-G{\underline{\varphi }}^{E}\right)}^{1-\beta }\circ {\left(U{\underline{\theta }}^{k}\right)}^{\beta -1}\right]}{\Gamma \;U^{T}\;\left[\underline{w}\circ {\underline{x}}^{\lambda }\circ {\left(\underline{x}-G{\underline{\varphi }}^{E}\right)}^{-\lambda }\circ {\left(U{\underline{\theta }}^{k}\right)}^{\lambda }\right]}\right)}^{\frac{1}{\alpha }}.$$

By replacing *U* according to Equation (32), and by noticing that $\Gamma \Gamma^{T}=\mathrm{diag}\left({\bar{\underline{\omega }}}^{E}\right)$, it results in the new update rule:

(42) $$▵{\underline{f}}^{k+1}\leftarrow ▵{\underline{f}}^{k}\circ {\bar{\underline{\omega }}}^{E}\circ N_{{\underline{f}}^{k}},$$

where

(43) $$N_{{\underline{f}}^{k}}≜{\left(\frac{G^{T}\left[\underline{w}\circ {\underline{x}}^{\lambda }\circ {\left(\underline{x}-G{\underline{\varphi }}^{E}\right)}^{1-\beta }\circ {\left(G▵{\underline{f}}^{k}\right)}^{\beta -1}\right]}{G^{T}\left[\underline{w}\circ {\underline{x}}^{\lambda }\circ {\left(\underline{x}-G{\underline{\varphi }}^{E}\right)}^{-\lambda }\circ {\left(G▵{\underline{f}}^{k}\right)}^{\lambda }\right]}\right)}^{\frac{1}{\alpha }}.$$

Similarly to [35], we derive the update rules by writing the matrix form of Equation (43), which completes the proof. □

Appendix A proposes the update rules for the problem (29). These rules are almost similar to those introduced above as they present some differences in the multiplicative mask. We show in Appendix A that the update rules proposed in the main part of this paper extend the ones proposed in Appendix A by iteratively updating the weights.

Update rules for *G* correspondto an unconstrained αβ-WNMF driven by Equation (12) since no information is available on *G*. Their validity is only guaranteed within the convex domain, i.e., for β∈[min(1,1−α);max(1,1−α)]. Outside this domain, some additional assumptions on the reconstructed data are needed to ensure the local convexity property [23]. As we chose to set α and β so that they belong to area 2 in Figure 3, the convexity domain reduces the possible area to the intersection between area 2 and the half-plane β≤1.

### 5.2. Normalization Procedures

In the considered application, the rows of the profile matrix are summed to one. This case is different from the one encountered in hyperspectral unmixing [44]—since our constraint cannot be split into independent vectorial subproblems—and in mobile sensor calibration [13] as the sum-constraint is only approximately satisfied in the latter. As a consequence, in our previous work [32,35], we used to normalize the matrices *G* and *F* in each iteration, after estimating them. We reformulate these steps below (see Section 5.2.1) while we investigate an alternative normalization procedure in Section 5.2.2. They are introduced in the framework of the above approach but the rules may be applied to our previous work [32,35] as well.

#### 5.2.1. Classical Normalization

Let us define $\tilde{F}$ as the normalized profile matrix and $\tilde{G}$ the corresponding scaled contribution matrix. In order to hold the sum-to-one property, Lantéri et al. [14] proposed a change of variables under the form (please note that the normalization constraint can also be solved as a penalization term in the NMF problem formulation [13]. This setting is interesting when the sum constraint is approximately satisfied, which is not the case for the considered application).

(44) $${\tilde{F}}_{ij}=\frac{F_{ij}}{\sum_{j=1}^{m}F_{ij}},$$

which may be written under matrix form as

(45) $$\tilde{F}=\frac{F}{F\;\cdot \;1_{mm}}.$$

This equation enables to normalize the rows of *F* whereas the symmetric version enables to scale the columns of *G* correspondingly, i.e.,

(46) $$\tilde{G}=G\circ \left[1_{nm}\;\cdot \;F^{T}\right].$$

The product $\tilde{G}\;\cdot \;\tilde{F}$ then reads

(47) $$\tilde{G}\;\cdot \;\tilde{F}=\frac{F}{F\;\cdot \;1_{mm}}\;\cdot \;\left(G\circ [1_{nm}\;\cdot \;F^{T}]\right),$$

which results in the expression of its general entry:

(48) $${\left(\tilde{G}\;\cdot \;\tilde{F}\right)}_{ij}=\sum_{k}\frac{G_{ik}}{\sum_{l}F_{kl}}\;\cdot \;F_{ki}\;\cdot \;\sum_{l}F_{kl}=\sum_{k}G_{ik}\;F_{ki}={\left(G\;\cdot \;F\right)}_{ij}.$$

This means that the matrix product is maintained throughout the normalization process. Since the cost function to minimize only depends on this product, this property ensures the same decrease as in the unconstrained case within iterations.

The normalized expression of *F*—denoted $\tilde{F}$—at iteration k+1 then reads

(49) $${\tilde{F}}^{k+1}\leftarrow \frac{\Phi^{E}\circ \Omega^{E}+\Delta {\tilde{F}}^{k}\circ {\bar{\Omega }}^{E}\circ R_{F}^{\alpha ,\beta }}{\left[\Phi^{E}\circ \Omega^{E}+\Delta {\tilde{F}}^{k}\circ {\bar{\Omega }}^{E}\circ R_{F}^{\alpha ,\beta }\right]\;\cdot \;1_{mm}},$$

where

(50) $$R_{F}^{\alpha ,\beta }≜\left\{\begin{array}{cc} M_{F}^{\alpha ,\beta }\left(G,F\right) & \mathrm{for}\mathrm{Problem}\;(29),\\ N_{F}^{\alpha ,\beta }\left(G,F\right) & \mathrm{for}\mathrm{Problem}\;(27), \end{array}\right.$$

and where $▵{\tilde{F}}^{k}$ stands for the free part of the normalized matrix $\tilde{F}$ defined by $▵{\tilde{F}}^{k}≜{\tilde{F}}^{k}\circ {\bar{\Omega }}^{E}$. Noticing that

(51) $$▵{\tilde{F}}^{k}\circ {\bar{\Omega }}^{E}={\tilde{F}}^{k}\circ {\bar{\Omega }}^{E}$$

we express Equation (49) with respect to ${\tilde{F}}^{k}$:

(52) $${\tilde{F}}^{k+1}\leftarrow \frac{\Omega^{E}\circ \Phi^{E}+{\tilde{F}}^{k}\circ {\bar{\Omega }}^{E}\circ R_{F}^{\alpha ,\beta }}{\left[\Omega^{E}\circ \Phi^{E}+{\tilde{F}}^{k}\circ {\bar{\Omega }}^{E}\circ R_{F}^{\alpha ,\beta }\right]\;\cdot \;1_{mm}}.$$

Similarly, we derive the update rules for ${\tilde{G}}^{k+1}$, i.e.,

(53) $${\tilde{G}}^{k+1}\leftarrow {\tilde{G}}^{k}\circ N_{G}^{\alpha ,\beta }\left({\tilde{G}}^{k},F^{k+1}\right)\circ \left[1_{nm}\;\cdot \;{\left(\Omega^{E}\circ \Phi^{E}+{\tilde{F}}^{k}\circ {\bar{\Omega }}^{E}\circ R_{F}^{\alpha ,\beta }\right)}^{T}\right],$$

where $N_{G}^{\alpha ,\beta }\left({\tilde{G}}^{k},F^{k+1}\right)$—defined in Equation (14)—is computed with the unnormalized matrix $F^{k+1}$ which reads

(54) $$F^{k+1}=\Omega^{E}\circ \Phi^{E}+{\tilde{F}}^{k}\circ {\bar{\Omega }}^{E}\circ R_{F}^{\alpha ,\beta }.$$

Equations (52) and (53) thus provide the update rules for our first normalized and constrained WNMF method denoted αβ-N${}_{1}$-constrained and weighted NMF (CWNMF) below. Although the set profiles are lost within iterations due to the normalization process, we noticed in preliminary tests that they were recovered asymptotically.

#### 5.2.2. Alternative Normalization

As an alternative, we now propose a second normalization which keeps the set constraints on *F* within iterations. Starting with Equation (30) that we normalize, it turns out that

(55) $$▵{\tilde{F}}^{k+1}\leftarrow \frac{\Delta {\tilde{F}}^{k}\circ {\bar{\Omega }}^{E}\circ R_{F}^{\alpha ,\beta }}{\left(\Delta {\tilde{F}}^{k}\circ {\bar{\Omega }}^{E}\circ R_{F}^{\alpha ,\beta }\right)\;\cdot \;1_{mm}}\circ \left(1_{pm}-\Phi^{E}\;\cdot \;1_{mm}\right),$$

where $\left(1_{p\times m}-\Phi^{E}\;\cdot \;1_{mm}\right)$ accounts for the matrix involving the sum of the free components for each source, and the other part of the expression represents the different proportions within the free profiles. Using the property (51), alternative update rules may be derived

(56) $${\tilde{F}}^{k+1}\leftarrow \Omega^{E}\circ \Phi^{E}+\frac{{\bar{\Omega }}^{E}\circ {\tilde{F}}^{k}\circ R_{F}^{\alpha ,\beta }}{\left[{\bar{\Omega }}^{E}\circ {\tilde{F}}^{k}\circ R_{F}^{\alpha ,\beta }\right]1_{mm}}\circ \left(1_{pm}-\Phi^{E}\;\cdot \;1_{mm}\right).$$

This normalization keeps the constraints verified within iterations but may move along directions different from the steepest descent direction. During this process, the contribution matrix does not require a scale factor as in the first method since the scale factor is only applied to the free parameters of *F*. We then estimate $G^{k+1}$ using the unconstrained rules defined in Equations (12) and (14). The update rules (12) and (56) are associated with our second normalized and constrained WNMF method denoted αβ-N${}_{2}$-CWNMF below.

#### 5.2.3. Description of Algorithm Acronyms

We proposed above some update rules for two methods for normalized and constrained WNMF. However, we also proposed different update rules in Appendix A for which the above normalizations can be applied. As these methods minimize the divergence between GΔF and the Residual $X-G\Phi^{E}$, we add a “-R” to their acronym. Table 3 outlines the necessary information for each method. The pseudo code for αβ-N${}_{x}$-CWNMF(-R) methods is shown in Algorithm 1.

**Algorithm 1**αβ-N${}_{x}$-constrained weighted non-negative matrix factorization (CWNMF) residual (-R) method. i←0 **while**
i≤N
**do**  Update *F* at fixed *G* according to Equation (52) or (56)  Update *G* at fixed *F* according to Equation (12) or (53)  i←i+1 **end while**

### 5.3. Bound-Constrained Normalized and Weighted αβ-NMF

We now focus on problem (25) which involves several kinds of constraints which should coexist simultaneously. To our knowledge, only Lin [45] deals with bound constraints and proposes to adapt the stepsize of projected gradient techniques in order to both decrease the cost function while holding the constraints. However, the work was devoted to bound constraints only, and his solution does not suit our problem with normalization.

As explained above, we propose to tackle them by applying a projection onto the admissible domain. Bound constraints act as safety barriers which prevent unrealistic solutions. However, the combination of normalization and projection should be applied in a predefined order. We thus propose below two structures:

1. the bound constraint projection followed by a normalization stage,
2. or the normalization followed by the projection.

#### 5.3.1. Informed NMF with Bound Constraints and Normalization

In this subsection, we consider update rules for N${}_{2}$-CWNMF methods. The same kind of procedure should be done for N${}_{1}$-CWNMF approaches proposed above. We assume that we get at iteration *k* a normalized matrix ${\tilde{F}}^{k}$ and an unscaled (indeed, no scaling is applied on *G* in N${}_{2}$-CWNMF, as explained in Section 5.2.2) contribution matrix $G^{k}$. Combining Equations (24) and (54) provide

(57) $$F^{k+1}=\Phi^{E}\circ \Omega^{E}+\Delta {\tilde{F}}^{k}\circ {\bar{\Omega }}^{E}\circ {\bar{\Omega }}^{I}\circ R_{F}^{\alpha ,\beta }+\Delta {\tilde{F}}^{k}\circ {\bar{\Omega }}^{E}\circ \Omega^{I}\circ R_{F}^{\alpha ,\beta },$$

which may be simplified by using Equation (51), i.e.,

(58) $$F^{k+1}=\Phi^{E}\circ \Omega^{E}+{\tilde{F}}^{k}\circ {\bar{\Omega }}^{E}\circ {\bar{\Omega }}^{I}\circ R_{F}^{\alpha ,\beta }+{\tilde{F}}^{k}\circ {\bar{\Omega }}^{E}\circ \Omega^{I}\circ R_{F}^{\alpha ,\beta }.$$

Applying the bound constraint then consists of

(59) $$F^{k+1}=\Phi^{E}\circ \Omega^{E}+{\tilde{F}}^{k}\circ {\bar{\Omega }}^{E}\circ {\bar{\Omega }}^{I}\circ R_{F}^{\alpha ,\beta }+P_{\Omega^{I}}\left({\tilde{F}}^{k}\circ {\bar{\Omega }}^{E}\circ R_{F}^{\alpha ,\beta }\right),$$

where $P_{\Omega^{I}}\left(.\right)$ is the projection operator defined by

(60) $$P_{\Omega^{I}}\left(U\right)≜\left\{\begin{array}{cc} \Omega^{I}\circ \Phi^{I-} & \mathrm{if}\;\Omega^{I}\circ U⪯\Phi^{I-},\\ \Omega^{I}\circ \Phi^{I+} & \mathrm{if}\;\Omega^{I}\circ U⪰\Phi^{I+},\\ \Omega^{I}\circ U & \mathrm{otherwise}. \end{array}\right.$$

The second normalization proposed in Section 5.2.2 consists of scaling the free part without changing the set components, which reads

(61) $${\tilde{F}}^{k+1}=\Phi^{E}\circ \Omega^{E}+\frac{{\tilde{F}}^{k}\circ {\bar{\Omega }}^{E}\circ {\bar{\Omega }}^{I}\circ R_{F}^{\alpha ,\beta }+P_{\Omega^{I}}\left({\tilde{F}}^{k}\circ {\bar{\Omega }}^{E}\circ R_{F}^{\alpha ,\beta }\right)}{\left({\tilde{F}}^{k}\circ {\bar{\Omega }}^{E}\circ {\bar{\Omega }}^{I}\circ R_{F}^{\alpha ,\beta }+P_{\Omega^{I}}\left({\tilde{F}}^{k}\circ {\bar{\Omega }}^{E}\circ R_{F}^{\alpha ,\beta }\right)\right)\;\cdot \;1_{mm}}\circ \left(1_{pm}-\Phi^{E}1_{mm}\right).$$

This rule keeps the sum-to-one constraint and the set values. The bound constraints may be lost within because of the normalization but were found to be asymptotically recovered in our tests. The associated updates for *G* follows the unconstrained ones and it has not to be corrected by a scale factor, i.e.,

(62) $$G^{k+1}=G^{k}\circ N_{G}^{\alpha ,\beta }\left(G^{k},{\tilde{F}}^{k}\right),$$

where $N_{G}^{\alpha ,\beta }\left(G^{k},{\tilde{F}}^{k}\right)$ has been introduced in Equation (14). The rules (61) and (62)—associated to our informed NMF approach named αβ-BN${}_{2}$-CWNMF—do not ensure the cancellation of the gradient of Equation (38) along iterations but they preserve two set of constraints among the three ones. Let us recall that the approach using the first proposed normalization—denoted αβ-BN${}_{1}$-CWNMF—can be derived with the same strategy. The pseudo code for αβ-BN${}_{1}$-CWNMF method is shown in Algorithm 2.

**Algorithm 2**αβ-BN${}_{1}$-CWNMF method i←0 **while**
i≤N
**do**  Update *F* at fixed *G* according to Equation (61)  Update *G* at fixed *F* according to Equation (62)  i←i+1 **end while**

#### 5.3.2. Informed NMF with Normalization and Bound Constraints

The same procedure as above should be applied in the reverse order so that bound projection is applied as the last step of an iteration. When applied to Equation (58), the second normalization provides

(63) $${\tilde{F}}^{k+1}=\Omega^{E}\circ \Phi^{E}+\left(1_{pm}-\Phi^{E}\;\cdot \;1_{mm}\right)\circ \left(\frac{{\bar{\Omega }}^{E}\circ {\bar{\Omega }}^{I}\circ F^{k}\circ R_{F}^{\alpha ,\beta }}{\left[{\bar{\Omega }}^{E}\circ F^{k}\circ R_{F}^{\alpha ,\beta }\right]1_{mm}}+\frac{{\bar{\Omega }}^{E}\circ \Omega^{I}\circ F^{k}\circ R_{F}^{\alpha ,\beta }}{\left[{\bar{\Omega }}^{E}\circ F^{k}\circ R_{F}^{\alpha ,\beta }\right]1_{mm}}\right).$$

The projection stage then leads to the unnormalized profile

(64) $$F^{k+1}=\Omega^{E}\circ \Phi^{E}+\frac{F^{k}\circ {\bar{\Omega }}^{E}\circ {\bar{\Omega }}^{I}\circ R_{F}^{\alpha ,\beta }}{\left(F^{k}\circ {\bar{\Omega }}^{E}\circ R_{F}^{\alpha ,\beta }\right)\;\cdot \;1_{mm}}\circ \left(1_{pm}-\Phi^{E}\;\cdot \;1_{mm}\right)+P_{\Omega^{I}}\left(\frac{F^{k}\circ {\bar{\Omega }}^{E}\circ R_{F}^{\alpha ,\beta }}{\left(F^{k}\circ {\bar{\Omega }}^{E}\circ R_{F}^{\alpha ,\beta }\right)\;\cdot \;1_{mm}}\circ \left(1_{pm}-\Phi^{E}\;\cdot \;1_{mm}\right)\right).$$

Equations (62) and (64) account for the update rules in this last method, denoted as αβ-N${}_{2}$B-CWNMF. The pseudo code for αβ N${}_{2}$B-CWNMF method is shown in Algorithm 3.

**Algorithm 3**αβ-N${}_{2}$B-CWNMF method i←0 **while**
i≤N
**do**  Update *F* at fixed *G* according to Equation (64)  Update *G* at fixed *F* according to Equation (62)  i←i+1 **end while**

Please notice that only set and bound constraints are checked within iterations. Convergence towards a limit point ensures that limit matrices keep all the desired properties. As explained above, the same procedure with our first considered normalization may be applied, thus yielding an approach named αβ-N${}_{1}$B-CWNMF.

## 6. Experimental Results

In this section, the enhancement provided by our methods are investigated in both simulations and a real data campaign. In these tests, we aim to identify the sources (by their chemical profile) contributing to the total atmospheric suspended PM as well as to quantify their contribution. In both the simulations and the real dataset, we consider atmospheric particles with diameter equal to or below 10 μm (PM${}_{10}$). In practice, these particles are trapped in a filter which is changed every 24 h. Each filter is then analyzed by chemists who derive the masses of several chemical species of interest for the considered application, i.e., for evaluating the impact of marine traffic on air pollution in a port city. Species under study are divided into 16 metal tracers—i.e., Al, Cr, Fe, Mn, P, Sr, Ti, Zn, V, Ni, Co, Cu, Cd, Sb, La, and Pb—8 water soluble ionic species—i.e., ${\mathrm{Na}}^{+}$, ${\mathrm{NH}}_{4}^{+}$, $K^{+}$, ${\mathrm{Mg}}^{2+}$, ${\mathrm{Ca}}^{2+}$, ${\mathrm{Cl}}^{-}$, ${\mathrm{NO}}_{3}^{-}$, and ${\mathrm{SO}}_{4}^{2-}$—carbon compounds—either organic (OC) or elementary (EC)—levoglucosan, and polyols.

In all these experiments, except when we tested the influence of these parameters, we set the values of α and β to 0.6 and 0.9, respectively. Indeed, such a couple of value lies in the recommended area 2 defined in Figure 3. Moreover, we found in preliminary tests that these values of α and β provided a better performance. As a consequence, we do not make them vary in the remainder of this section.

Moreover, the signal-to-noise ratio (SNR) enabled us to evaluate the data set and is defined as:

(65) $$\mathrm{SNR}\left(X\right)=\frac{1}{m}\sum_{j=1}^{m}\;SNR_{j}\left({\underline{x}}_{j}\right)=\frac{1}{m}\sum_{j=1}^{m}10\;\log_{10}\frac{\sum_{i=1}^{n}x_{ij}^{2}}{\sum_{i=1}^{n}e_{ij}^{2}},$$

where $x_{ij}$ and $e_{ij}$ stand for the (i,j)-th non-noisy data and the individual noise. This index is widely used in the literature [46].

### 6.1. Realistic Simulations 

From the validated profile and contribution matrices obtained during the real campaign [47], simulation data were built by taking into account the individual uncertainty provided by the real campaign. In these simulations, the data matrix *X* thus consisted of a 278×28 matrix—which correspond to the chemical composition (28 species) of 278 PM samples—associated with individual uncertainties, which are those provided by the chemical analysis.

In addition, we also considered several cases with outliers. It is assumed that outliers come from an additional positive individual contamination.

The mathematical model of the outliers was driven by a random vector idx_outliers including the locations of the outliers in the data matrix. For these locations, a multiplicative model was used depending of the trial number *i* (between 1 and 400).

(66) $$X1\left(idx\_outliers\right)=\left(1+\frac{i}{ratio}\right)*X\left(idx\_outliers\right),$$

where X1 (resp. *X*) accounts for the with outliers noiseless data (resp. the without outliers noiseless data). The variable ratio is a parameter which may be tuned in order to get a SNR after outliers ranging from 15 dB to 70 dB. In our tests, the outlier deviation increased with the trial number *i*. In other words, for low trial number, the multiplicative factor remained close to 1 in order to keep large. The effect of such outliers essentially depends on the location of the outliers. Indeed, if an outlier acts on a large entry of the data matrix, its impact on the SNR will be greater.

Then, a noise has to mimic the chemical measurement process. The chemical measurement process only gives a concentration value together with an uncertainty. So, every value within this interval is equally possible. A uniform noise which is designed on a limited support was proposed. This support may be truncated on the left side if the uncertainty is greater than the corresponding data.

Among the 278 samples, 10 and 20 outliers were considered. Practically, we noticed that the signal-to-noise ratio (SNR) index then dropped in the worst case by 4 dB if the set of 20 outliers is taken into consideration with respect to the no outlier case.

#### 6.1.1. Source Profiles

In this study, 10 sources are highlighted. Among them, some of them are purely natural or purely anthropogenic but some of them became anthropised. Table 4 describes major species present in each source profile. Other species than those listed in the corresponding source profile may be considered as negligible. Please note that—as we here consider simulations—the real profile matrix is perfectly known and is provided in Table A1. Also, one should notice that each source profile is presented under a per mil notation, i.e., it sums to a thousand instead of 1 and the only difference is a scale factor equal to 1000.

#### 6.1.2. Equality Constraints

Equality constraints or set values enable to inform the algorithm about some entries of the profile matrix. This knowledge is taken into account by specifying matrices $\Omega^{E}$ and $\Phi^{E}$. These matrices are available in Appendix B. It is to be stressed that the only used knowledge here is the absence of some compounds in some source profiles. As a result, matrix $\Phi^{E}$ reduced to $0_{10\times 28}$. Then, it follows that our informed methods with residuals were identical to those without residual. As a consequence, we do not test the latter in the simulations below.

#### 6.1.3. Initialization

An approximate prior knowledge of *F* was used as a starting point for each informed NMF algorithm. Table A3 gathers the different entries used. Then, a weighted quadratic estimation of the initial contribution matrix *G* [31] was performed so that each method has the same initial factors.

#### 6.1.4. Performance Evaluation

Several performance indexes are available in the literature. However in this work, only the mixing-error ratio (MER) index [52] is considered (please note that while specifically designed for measuring the estimation accuracy of a mixing matrix, the MER may also be used as a signal-to-inteference ratio (SIR) when applied to the profile matrix, and more specifically to $F^{T}$). It was computed over each column of *G*. For each source, a scalar quantity $MER_{j}$ for source *j* expressed in dB may be obtained.

For one exact vector ${\underline{g}}_{j}$ and its estimate ${\hat{\underline{g}}}_{j}$, it is possible to write ${\hat{\underline{g}}}_{j}$ under the form

(67) $${\hat{\underline{g}}}_{j}={\hat{\underline{g}}}_{j}^{coll}+{\hat{\underline{g}}}_{j}^{orth},$$

where ${\hat{\underline{g}}}^{coll}$ and ${\hat{\underline{g}}}^{orth}$ are respectively colinear and orthogonal to the exact vector $\underline{g}$. This decomposition allows to express the MER of source *j*, denoted as $MER_{j}$, defined as,

(68) $$MER_{j}=10\;\log_{10}\frac{∥{\hat{\underline{g}}}_{j}^{coll}{∥}^{2}}{∥{\hat{\underline{g}}}_{j}^{orth}{∥}^{2}}.$$

Infinite values mean exact separation while 0 dB correspond to an angle equal to 45°. These values may be summed up into a vector which gathers the performance of each source. Generally, a global indicator is obtained by averaging each index over all sources, i.e.,

(69) $$MER=\frac{1}{p}\sum_{j=1}^{p}\;MER_{j}.$$

In all the cases under study, the MER (the results and the Matlab interpretation codes are already available at http://www-lisic.univ-littoral.fr/~delmaire/recherche.html) index [52] was represented as a function of the input SNR. In this study, intensive computations were performed with ten thousand iterations for each method over 400 tests. In our comparison, we dropped the PMF method as it is only available as a user interface (see https://www.epa.gov/air-research/positive-matrix-factorization-model-environmental-data-analyses) which prevents to compute several tests in a single command. Moreover, even for a single test, our expertise shows that PMF requires the uncertainties to be increased in order to perform a computation, but it did not make sense in this case. As a consequence, nine methods were selected and tested: among them, three are uninformed, two account for our informed methods with set values while the four remaining ones are our informed methods with bounds.

**Figure 4:**
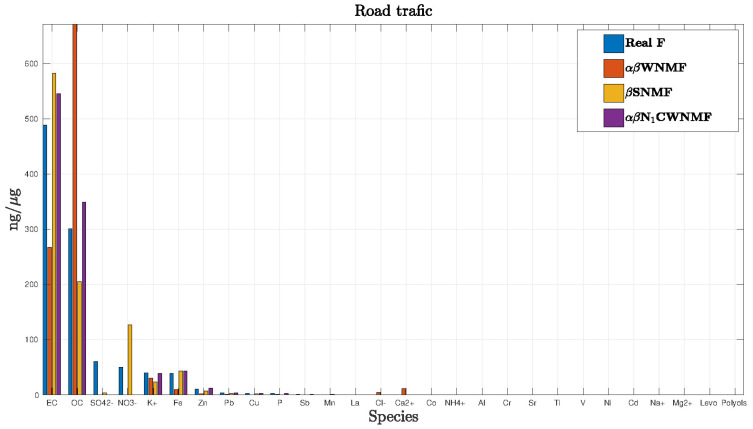
Estimation of the road traffic profile.

In order to get an idea, we chose to display the road traffic profile estimation in the case when input SNR is equal to 24 dB (Figure 4). Species were represented in descending order of the real profile. We could notice that for this source, αβ N${}_{1}$CWNMF appears better than other methods.

**Figure 5:**
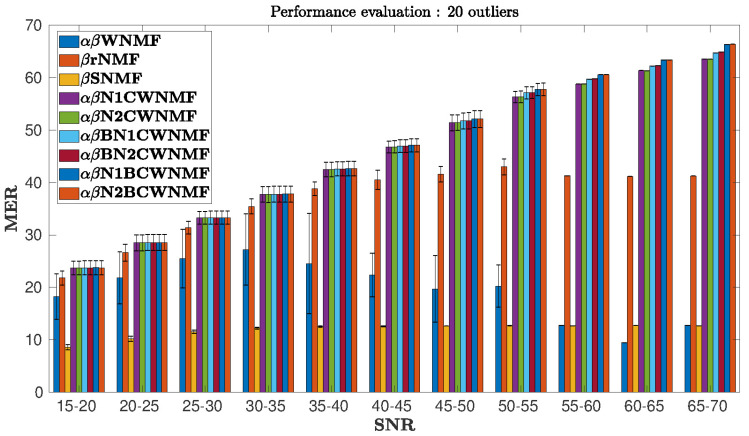
MER vs. input signal-to-noise ratio (SNR). The case with 20 outliers.

In our tests, the input SNR ranges from 15 to 70 dB. We decided to display only the performance of the methods for 20 outliers as shown in Figure 5 since the other tests provide similar results. The statistical performance is provided in Figure 5 by specifying the standard deviation in each slice of SNR and for each method.

Let us first analyze the enhancement provided by the non-informed NMF methods. One notice that the robust αβ-WNMF [23] performs very poorly in all cases. Its standard deviation appears very large for a wide range of SNRs. Besides, RNMF—which stands for a robust NMF method [18]—behaves correctly for low SNRs while its performance decreases surprisingly for large SNRs. Moreover, we experimented a sparse NMF (SNMF) method [53] including a β-divergence cost function together with L${}_{1}$ sparsity of one factor. We select one trial and test the performance for the parameter β ranging from 0 to 2. The optimal value β=0.5 has been selected over 400 trials for the case of 20 outliers. SNMF provides inconsistent solutions in every slice of SNR.

We analyzed the performance of our proposed informed methods. Let us firstly focus on both informed methods with set values which were experimented, i.e., the αβ-N1CWNMF and αβ-N2CWNMF methods. Their performance appeared to be very similar in all the simulations. In practice, their MER was approximately equal to the SNR in every input SNR slice, which was expected according to our experience in preliminary tests.

The four informed NMF methods with bound constraints behaved similarly, except in a few slices where the SNR is large. Indeed, in low SNR, they are slightly better than αβ-N1CWNMF (the gap is not visible due to the scale), but they outperform all the other tested methods as soon as the SNR becomes greater than 40 dB. The low gap in low SNR is essentially due to the fact that we inform *F* while the performance index is measured on *G*. In noisy tests—i.e., for a low SNR—the estimated matrix *G* does not benefit from the additional information on *F*, because of the important noise in *X*. However, we noticed an improvement on *F* for these tests, even if we cannot safely measure it, as the profiles might be correlated.

On the contrary, for medium and large SNR, the MER enhancement was significant for every bound-constrained informed NMF method. More precisely, αβ-N1BCWNMF and αβ-N2BCWNMF outperformed all the other methods with a significant gap as soon as the SNR increased.

We also explored in the synthetic example the use of a large range of α and β parameters within area 2 such that 0.5⪯α⪯1 and 0.5⪯β⪯1. We noticed that the MER index for αβ WNMF method was very sensitive to the choice of the α β parameters and also to the trial number. A successful tuning of these parameters was somehow difficult.

**Figure 6:**
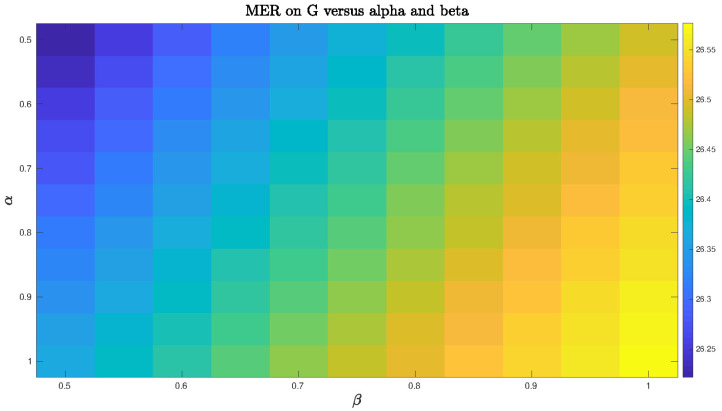
Mixing-error ratio (MER) index for N${}_{1}$ constrained and weighted NMF mixing-error ratio (CWNMF) vs. α and β.

On the other hand, we experiment the same operating conditions for N${}_{1}$CWNMF. We observe in Figure 6 that results are more stable than for the uninformed one. In this case, the choice of αβ appears quite insensitive but the method remains satisfactory.

**Figure 7:**
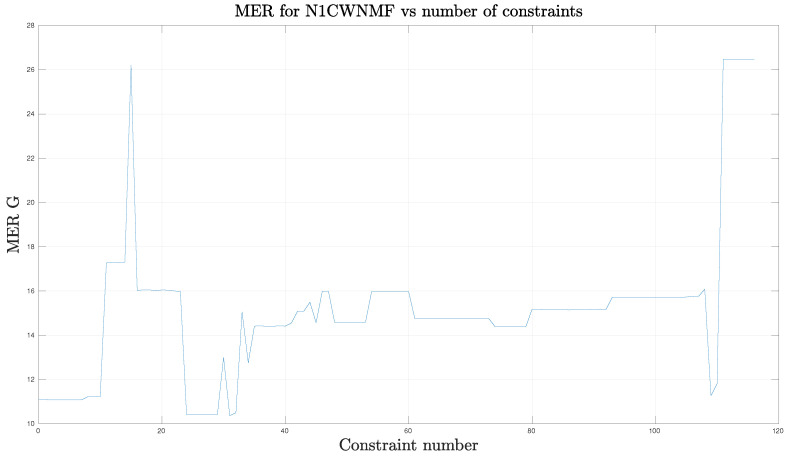
MER versus constraint number. The case with 20 outliers.

In addition, we could wonder how constraints affect the results. First, we potentially may use 117 set values and roughly 60 bound constraints. We decided to inspect the influence of dropping set values only. For that purpose, we progressively turned on one set value at a time for each column and according to the increasing order of the row index, until the 117 constraints were reached. We plot the MER performance according to the number of constraints in Figure 7.

Contrary to what should be expected, adding constraints may sometimes degrade the performance suddenly or conversely. There seems to be set of constraints which fit better to the situation. This conclusion is quite surprising and the design of appropriate constraints seems an open question.

To conclude, these methods provide a good performance in every situation and are thus better-suited for the considered application.

### 6.2. Real Data Case

The real data campaign was conducted by Dr C. Roche during her Ph.D. thesis [54], within the UCEIV laboratory (Université du Littoral Côte d’Opale). The first goal of this thesis was to study how much the shipping traffic in the English Channel, one of the most important in the world, can contribute to the atmospheric PM${}_{10}$ concentration in coastal area, such as the Hauts–de–France region. In her work, some characteristic species of maritime traffic emissions have been evidenced. Then, some flexible bound profiles and set profile entries were proposed. Using this knowledge, the challenge was to implement an informed NMF method—as those developed in [32]—in order to reconstruct the PM origin.

Contrary to [54], we here would like to drop some of the bound information and to test whether or not the new methods that we propose in this paper are still competitive.

#### 6.2.1. Context

A sampling campaign has been conducted using a Digitel DA80 sampler over a long period—i.e., 16 months—in Cape Gris–Nez and over a shorter period—i.e., three months—in the port of Calais, which enabled us to get 278 sample measurements. Cape Gris–Nez and Calais are two coastal sites in the eastern part of the English channel. The first one is a rural site whereas the port of Calais is the second busiest in passenger traffic in Europe with 10.8 million of passengers and over 80 arrivals and departures of ferries per day in average in 2014 [55].

**Figure 8:**
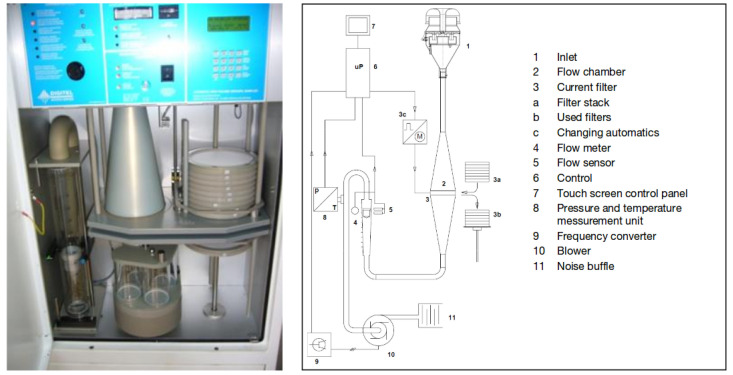
Digitel DA80 high volume sampler used for data acquisition (source of the right plot: Digitel).

The DA80 device (see Figure 8) is an equipment which is able to trap PM on filters, which are stored and a posteriori analyzed for chemical composition. A special sieve enabled us to select only PM${}_{10}$, i.e., PM whose diameter was lower than 10μm. The machine is also able to save wind conditions and time. The sampling period was chosen equal to 24 h. Along this period, meteorological conditions concentration levels were highly varying. Thus, after analyzing the filters, several data files were available to address the pollution source apportionment problem.

#### 6.2.2. Input Data

Appendix C provides operating conditions for the run which are performed. Based on the expert knowledge provided by chemists and on the information described in Table 4, a matrix $\Omega^{E}$—which defines 55 set value locations (among 278 profile entries)—is provided in Table A5. In the same way as in Section 6.1, the matrix $\Phi^{E}$ is equal to $0_{p\times m}$. In addition, the initial profile matrix is chosen by an expert and is provided in Appendix C.

#### 6.2.3. Results Evaluation

The results were obtained in the case of 10 identified sources and $10^{4}$ iterations for each method. The profiles under study are specified in Table 4. However, their estimation remains a difficult task for several reasons which listed hereafter:

- Data are corrupted by an unknown number of outliers. Their origin may be of various kinds, e.g., the presence of a new source which affects the data at some sparse moments.
- Data are very noisy. In particular, an additional overall pollution—whose level highly varies over time—can not be assigned to a particular source and can significantly decrease the overall SNR.
- Some source profiles may be geometrically close, only a few tracer species are able to distinguish them.

Even if a database with source profiles is available at http://source-apportionment.jrc.ec.europa.eu/Specieurope/sources.aspx, a universal profile for a given source does not exist. When comparing our result, they all appear highly consistent with the published one in the Specieurope database. We display the source profiles in a descending order of expected species (MPMPthis task was designed by the chemist co-authors of the paper). A correct source profile was then displayed as decreasing proportions from the left to the right of each figure. On the contrary, a large proportion on the right part of a profile plot implies that the estimation has partly failed.

**Figure 9:**
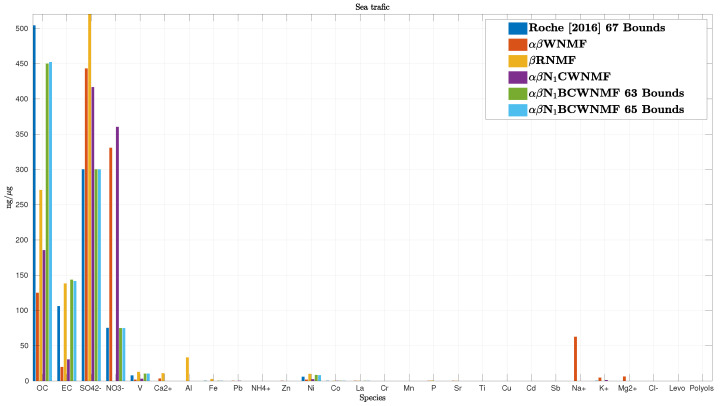
Estimation of the sea traffic source profile.

Among the 10 source profiles, some of them are well recovered. We only show in Figure 9 the estimated sea traffic source profile as it is difficult to recover. As mentioned above, it is expected that proportions are decreasing from the left to the right side of the figure. The order has been built based on ship profiles from the European database and from the literature [50,51,54]. To process these data, we compare the enhancement provided by two non-informed methods, i.e., the αβ-WNMF and the β-RNMF [18] and three informed methods, i.e., the method used in [54] and our methods αβ-N1CWNMF, and its bound-constrained extension αβ-N1BCWNMF. Other bound methods were dropped since they turn out in Section 6.1 to behave roughly similar to αβ-N1BCWNMF. Note that Roche [54] used 67 constraints while we only use 63 and 65 bound constraints in the tested bound-constrained informed αβ-NMF method, respectively.

It may be noticed that blind NMF methods, i.e., the αβ-WNMF and the β-RNMF, and our αβ-N1CWNMF method are overestimating ${\mathrm{SO}}_{4}^{2-}$ and ${\mathrm{NO}}_{3}^{-}$ species while underestimating OC and EC compounds. The estimated sea traffic profile thus appears not to be very realistic with these methods. Besides, bound-constrained WNMF methods behave similarly and report good estimations for major species. However, these estimations reach the proposed bounds for Fe, ${\mathrm{NO}}_{3}^{-}$, and ${\mathrm{SO}}_{4}^{2-}$ species among the 28 species under study. For example, ${\mathrm{SO}}_{4}^{2-}$ is limited by the maximum value provided in Table A4. Finally, these bound-constrained NMF methods outperform all the other methods for the sea traffic re-construction.

Using the estimation provided by each method, it is possible to reconstruct each species’ concentration, and especially the V and Ni compounds since they are tracers of the sea traffic activity [54]. In other words, the V and Ni species can only be found in the sea traffic source. Moreover, the ratio V over Ni is often assimilated to a value between 2 and 3 [51], and is found to be between 1.2 to 1.5 for the three bound-constrained WNMF methods, which is close to the expected ratio.

**Figure 10:**
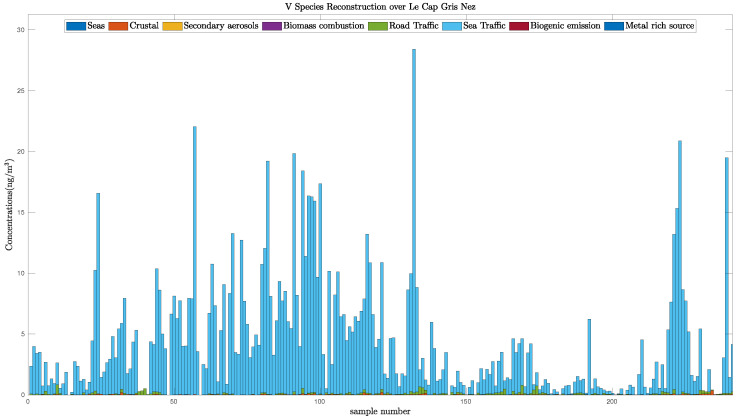
V species reconstruction over Cape Gris–Nez.

To confirm this fact, we plot the reconstruction of the V species in Figure 10. This shows that this compounds is mainly due to Sea Traffic. More than 98 per cent of the V species originates from the sea traffic source which is consistent with the chemist’s expectations.

## 7. Conclusions

In this paper, we tackled an informed non-negative matrix factorization problem where the profile matrix lives in a specific subspace. We proposed several informed NMF methods combining αβ-divergence and a specific structure of one matrix factor provided by the considered problem. This work extends our previous informed NMF [32]—assuming some values of one of the factor matrices to be known—by considering generalized divergences, and by leading to alternative update rules and normalization. The update rules may be viewed as projective multiplicative updates applied to a special structure of the profile matrix. The relevance of these extensions were shown on realistic simulations of natural and industrial PM source apportionment—with various input SNR conditions and various numbers of outliers—and on a real data case. In practice, these informed methods are more robust than blind NMF, and provide results which are consistent with the chemical expert, even in the presence several outliers. In future work, we will investigate new soft constraints to inform NMF and alternatives to multiplicative updates.

## Figures and Tables

**Table 1:** Properties of α-zoom.

α	$0<\frac{X_{\mathrm{ij}}}{{\hat{X}}_{\mathrm{ij}}}<1$	$\frac{X_{\mathrm{ij}}}{{\hat{X}}_{\mathrm{ij}}}>1$
α>1	small zoom	large zoom
α<1	large zoom	small zoom

**Table 2:** Weighting effect on the αβ-divergence.

α+β	$0<{\hat{X}}_{\mathrm{ij}}<1$	${\hat{X}}_{\mathrm{ij}}>1$
α+β<1	large weighting	small weighting
α+β>1	small weighting	large weighting

**Table 3:** Our different non-negative matrix factorization (NMF) methods with normalization.

Acronym	*F*	*G*	Mask on *F*	Mask on *G*
αβ-N${}_{1}$-CWNMF-R	Equation (52)	Equation (53)	$M_{F}^{\alpha ,\beta }\left({\tilde{G}}^{k},{\tilde{F}}^{k}\right)$	$N_{G}^{\alpha ,\beta }\left({\tilde{G}}^{k},F^{k+1}\right)$
αβ-N${}_{1}$-CWNMF	Equation (52)	Equation (53)	$N_{F}^{\alpha ,\beta }\left({\tilde{G}}^{k},{\tilde{F}}^{k}\right)$	$N_{G}^{\alpha ,\beta }\left({\tilde{G}}^{k},F^{k+1}\right)$
αβ-N${}_{2}$-CWNMF-R	Equation (56)	Equation (12)	$M_{F}^{\alpha ,\beta }\left(G^{k},{\tilde{F}}^{k}\right)$	$N_{G}^{\alpha ,\beta }\left(G^{k},{\tilde{F}}^{k+1}\right)$
αβ-N${}_{2}$-CWNMF	Equation (56)	Equation (12)	$N_{F}^{\alpha ,\beta }\left(G^{k},{\tilde{F}}^{k}\right)$	$N_{G}^{\alpha ,\beta }\left(G^{k},{\tilde{F}}^{k+1}\right)$

**Table 4:** Features of the different source profiles.

Profiles	Type	Major Species	References
Sea salts	Natural	${\mathrm{Cl}}^{-}$, ${\mathrm{Na}}^{+}$, ${\mathrm{SO}}_{4}^{2-}$, ${\mathrm{Mg}}^{2+}$, $K^{+}$, ${\mathrm{Ca}}^{2+}$, Sr	[48]
Crustal dust	Natural	Al, ${\mathrm{Ca}}^{2+}$, Fe, $K^{+}$, OC, Ti, ${\mathrm{NO}}_{3}^{-}$, ${\mathrm{Na}}^{+}$	[49]
Primary biogenic emission	Natural	OC, EC, Polyols, P	[50]
Aged sea salts	Anthropised	${\mathrm{NO}}_{3}^{-}$, ${\mathrm{Na}}^{+}$, ${\mathrm{SO}}_{4}^{2-}$, ${\mathrm{Mg}}^{2+}$, $K^{+}$, OC, ${\mathrm{Ca}}^{2+}$, Sr,${\mathrm{Cl}}^{-}$	[50]
Secondary nitrates	Anthropised	${\mathrm{NO}}_{3}^{-}$, OC, ${\mathrm{NH}}_{4}^{+}$, EC, ${\mathrm{Ca}}^{2+}$, Fe, Zn, Cu	[50]
Secondary sulfates	Anthropised	${\mathrm{SO}}_{4}^{2-}$, ${\mathrm{NH}}_{4}^{+}$, OC, ${\mathrm{Ca}}^{2+}$, $K^{+}$, Fe, Pb, Zn	[49]
Biomass combustion	Anthropogenic	OC, EC, Levoglucosan, ${\mathrm{NO}}_{3}^{-}$, $K^{+}$, Zn	[50]
Road traffic	Anthropogenic	EC, OC, ${\mathrm{NO}}_{3}^{-}$, Cu, Sb, Zn, Fe	[50]
Sea traffic	Anthropogenic	OC, EC, V, Ni, Co, ${\mathrm{SO}}_{4}^{2-}$, ${\mathrm{NH}}_{4}^{+}$, ${\mathrm{NO}}_{3}^{-}$	[50,51]
Rich metal source	Anthropogenic	Fe, Al, Cr, Pb, Zn, Mn	[50]

## Abbreviations

The following abbreviations are used in this manuscript:

CWNMF	Constrained and weighted NMF
KKT	Karush–Kuhn Tucker
EC	Elementary carbon
MER	Mixing-error ratio
MM	Majorization-minimization
NMF	Non-negative matrix factorization
OC	Organic carbon
PM	Particulate matter
RNMF	Robust NMF
SIR	Signal-to-interference ratio
SNR	Signal-to-noise ratio
WNMF	Weighted NMF

## Appendix A. Update Rules for Problem (29)

In this appendix, we aim to develop update rules for problem (29), for which we drop the sum-to-one constraint. The structure of the proof follows the same steps as in Section 5.1 within a MM strategy.

**Proposition** **A1.**
*Update rules for the free part of the profile matrix are*

(A1) $$▵F^{k+1}\leftarrow ▵F^{k}\circ {\bar{\Omega }}^{E}\circ M_{F}^{\alpha ,\beta }\left(G^{k},F^{k}\right),$$

*where*

(A2) $$M_{F}^{\alpha ,\beta }\left(G,F\right)≜{\left[\frac{G^{T}\left(W\circ {\left(X-G\Phi^{E}\right)}^{\alpha }\circ {\left(G(F^{k}\circ {\bar{\Omega }}^{E})\right)}^{\beta -1}\right)}{G^{T}\left(W\circ {\left(G(F^{k}\circ {\bar{\Omega }}^{E})\right)}^{\lambda }\right)}\right]}^{\left(\frac{1}{\alpha }\right)}.$$

**Proof.** We focus on a column of the data and drop hereafter the index *i* for the vectors $▵{\underline{f}}_{i}$, ${\underline{\varphi }}_{i}^{E}$, ${\underline{\theta }}_{i}$, and for the matrix $\Gamma_{i}$. Let us define the residual vector $\underline{r}$ as

(A3) $$\underline{r}≜\underline{x}-G{\underline{\varphi }}^{E}.$$

Combining Equations (21) and (32) leads to

(A4) $$D_{\underline{w}}^{\alpha ,\beta }\left(\underline{r}∥G\Delta \underline{f}\right)=D_{\underline{w}}^{\alpha ,\beta }\left(\underline{r}∥U\underline{\theta }\right)=\sum_{i}\;w_{i}\;r_{i}^{\alpha +\beta }\;\cdot \;h^{\alpha ,\beta }\left(\frac{\sum_{j}u_{ij}\theta_{j}}{r_{i}}\right),$$

where *U* is defined in Equation (32), and where $h^{\alpha ,\beta }\left(z\right)$ has been defined in Equation (35). Using the convexity of $h^{\alpha ,\beta }\left(z\right)$ for z≥0 and β∈[min(1,1−α);max(1,1−α)] [23], Jensen inequality may be applied once, resulting in:

(A5) $$h^{\alpha ,\beta }\left(\frac{\sum_{j}u_{ij}\theta_{j}}{r_{i}}\right)\leq \sum_{j}\frac{u_{ij}\theta_{j}^{k}}{\sum_{l}u_{il}\theta_{l}^{k}}\;h^{\alpha ,\beta }\left(\frac{\theta_{j}\sum_{l}u_{il}\theta_{l}^{k}}{r_{i}\theta_{j}^{k}}\right),$$

where the superscript *k* is the current iteration number and $\theta_{j}$ is the *j*-th element of the free parameters vector $\underline{\theta }$ introduced in (20). Equation (A4) together with Equation (A5) yield the majoring function

(A6) $$H_{1,w}^{\alpha ,\beta }\left(\theta_{j},\theta_{j}^{k}\right)=\sum_{i}w_{i}\;r_{i}^{\alpha +\beta }\;\sum_{j}\frac{u_{ij}\;\theta_{j}^{k}}{\sum_{l}u_{il}\;\theta_{l}^{k}}\;\cdot \;h^{\alpha ,\beta }\left(\frac{\theta_{j}\;\sum_{l}u_{il}\;\theta_{l}^{k}}{r_{i}\;\theta_{j}^{k}}\right).$$

Cancelling its gradient $\frac{\partial H_{1,w}^{\alpha ,\beta }\left(\theta_{j},\theta_{j}^{k}\right)}{\partial \theta_{j}}$ leads to

(A7) $${\left(\frac{\theta_{j}}{\theta_{j}^{k}}\right)}^{\alpha }=\frac{\sum_{i}w_{i}\;\;u_{ij}\;\;r_{i}^{\alpha }\;{\left(\sum_{l}u_{il}\theta_{l}^{k}\right)}^{\beta -1}}{\sum_{i}w_{i}u_{ij}\;\cdot \;{\left(\sum_{l}u_{il}\theta_{l}^{k}\right)}^{\lambda }},$$

which reads in vector form:

(A8) $${\left(\frac{\underline{\theta }}{{\underline{\theta }}^{k}}\right)}^{\alpha }=\frac{U^{T}\left[\underline{w}\circ {\underline{r}}^{\alpha }\circ {\left(U{\underline{\theta }}^{k}\right)}^{\beta -1}\right]}{U^{T}\left[\underline{w}\circ {\left(U{\underline{\theta }}^{k}\right)}^{\lambda }\right]}.$$

By combining the definition (21) with the above relationship, we derive the expression of one column of the matrix ▵F.

(A9) $$\frac{▵{\underline{f}}^{k+1}}{▵{\underline{f}}^{k}}={\left[\frac{\Gamma \;U^{T}\;\left[\underline{w}\circ {\underline{r}}^{\alpha }\circ {\left(U{\underline{\theta }}^{k}\right)}^{\beta -1}\right]}{\Gamma \;U^{T}\;\left[\underline{w}\circ {\left(U{\underline{\theta }}^{k}\right)}^{\lambda }\right]}\right]}^{\frac{1}{\alpha }}.$$

By replacing *U* according to Equation (32), and by noticing that $\Gamma \Gamma^{T}=diag\left({\bar{\underline{\omega }}}^{E}\right)$, it results in the new update rule:

(A10) $$▵{\underline{f}}^{k+1}\leftarrow ▵{\underline{f}}^{k}\circ {\bar{\underline{\omega }}}^{E}\circ M_{{\underline{f}}^{k}}^{\alpha ,\beta },$$

where $M_{{\underline{f}}^{k}}^{\alpha ,\beta }$ accounts for

(A11) $$M_{{\underline{f}}^{k}}^{\alpha ,\beta }≜{\left(\frac{G^{T}\left[\underline{w}\circ {\underline{r}}^{\alpha }\circ {\left(G▵{\underline{f}}^{k}\right)}^{\beta -1}\right]}{G^{T}\left[\underline{w}\circ \circ {\left(G▵{\underline{f}}^{k}\right)}^{\lambda }\right]}\right)}^{\frac{1}{\alpha }}.$$

Combining Equations (18) and (22) enables to express

(A12) $$\Delta F^{k}=\Delta F^{k}\circ {\bar{\Omega }}^{E}=F^{k}\circ {\bar{\Omega }}^{E}.$$

It yields the update rule by combining the matrix form of Equations (A11) and (A12), i.e.,

(A13) $$F^{k+1}\leftarrow \Phi^{E}+F^{k}\circ {\bar{\Omega }}^{E}\circ M_{F}^{\alpha ,\beta }\left(G^{k},F^{k}\right),$$

where $M_{F}^{\alpha ,\beta }\left(G,F\right)$ is defined in Equation (A2). This relation completes the proof. □

As explained in Section 5.1, it should be noticed the update rules for both variants of the weighted αβ-divergence cost function provide almost similar update rules. In fact, $N_{F}^{\alpha ,\beta }\left(G,F\right)$ in Equation (31) and $M_{F}^{\alpha ,\beta }\left(G,F\right)$ in Equation (A2) are the same if we replace *W* in the latter expression by

(A14) $$W≜W^{\prime }\circ X^{\lambda }\circ {\left(X-G\Phi^{E}\right)}^{-\lambda }.$$

As *G* is updated at each NMF iteration, the value of *W* in Equation (A14) varies at each iteration, which means that the update rules proposed in the main part of this paper extend the one proposed in Appendix A by iteratively updating the weights.

## Appendix B. Operating Conditions for the Simulations

Table A1 specifies the different entries of the true profile matrix for simulations.

Prior information is provided through the specification of both $\Omega^{E}$ and $\Phi^{E}$. We choose to select only set values which specify the absence of some species in the profile matrix. As a consequence, $\Phi^{E}$ is equal to $\Phi^{E}=0_{p\times m}$. The position of these known zero entries are provided in Table A2.

The chemists are able to provide an initial profile matrix which is given in Table A3. It is to be noted that the same initial matrix is applied for both the informed and non informed methods. It should be noticed that the known zeros in *F* are initialized as 1.00 $\times \;10^{-11}$.

**Table A1:** Theoretical source profile used in the simulations.

Profiles	Al	Cr	Fe	Mn	P	Sr	Ti	Zn	V	Ni	Co	Cu	Cd	Sb
Sea	0.0019	0	0	0	2.5 $\times \;10^{-4}$	0.2034	0	0	0	0	0	0	0	0
Aged sea	0	7.2351 $\times \;10^{-5}$	0	0	0.5	0.4	1.877 $\times \;10^{-4}$	0	0	0	1.785 $\times \;10^{-4}$	1.7941 $\times \;10^{-5}$	0	0
Crustal	119.13	8.589 $\times \;10^{-5}$	77.35	1.782	3.0680	0.7846	8.9121	1.868	0.3503	0	0.0276	0.0081	0	0
nitrates	4.00 $\times \;10^{-3}$	2 $\times \;10^{-5}$	3.5	0.11	0.0749	0	0	0.7742	0	0	7.0408 $\times \;10^{-4}$	0.1	6.486 $\times \;10^{-3}$	0.01975
sulfate	0	5 $\times \;10^{-5}$	0	0.02825	0.05313	0	0	0.1334	0	0	0.003287	8.00 $\times \;10^{-6}$	0	0
Biomass	0.001	0	2.554	0.05527	0	1.016 $\times \;10^{-5}$	0	0.1415	0	0	0	0	0	0.0385
Road traffic	0	0	39.0414	0.1404	2.659	0	0	10.908	0	0	1.00 $\times \;10^{-8}$	2.7712	0	0.8964
Sea traffic	0.001147	1.2012 $\times \;10^{-4}$	0.1002	0	0	0.0217	9.42 $\times \;10^{-5}$	0	7.4920	5.5348	0.1829	1.752 $\times \;10^{-4}$	1.315 $\times \;10^{-6}$	0
Biogenic	0	0	0	0	14.528	0.04308	8.941 $\times \;10^{-4}$	0	0	0	0	0	0	5.2 $\times \;10^{-4}$
Metal	64.430	33.332	780.16	33	0.7	2	0	0	0	10	0.15	1.5	1.55	0
Bis	La	Pb	${\mathrm{Na}}^{+}$	${\mathrm{NH}}_{4}^{+}$	$K^{+}$	${\mathrm{Mg}}^{2+}$	${\mathrm{Ca}}^{2+}$	${\mathrm{Cl}}^{-}$	${\mathrm{NO}}_{3}^{-}$	${\mathrm{SO}}_{4}^{2-}$	OC	EC	Levo.	Polyols
Sea	0	0	297.03	0	10.71	32.75	9.183	581.02	0	69.08	0	0	0	0
Aged sea	0	0.1	280	0	4	30	10	1.00 $\times \;10^{2}$	395	150	30	0	0	0
Crustal	0.0594	0	1.8333 $\times \;10^{-4}$	4.36 $\times \;10^{-5}$	5	5	301.81	0	49.95	39.96	384.92	0	0	0
nitrates	7.178 $\times \;10^{-4}$	0.2075	0	216.26	3.2	0	0	1.21 $\times \;10^{-5}$	730.73	0	45	0	0	9.027 $\times \;10^{-11}$
sulfate	0	0.0729	0	260.83	4.43	0	0	8.66 $\times \;10^{-8}$	0	680.59	53.84	0	0	0
Biomass	0	0.1007	2.650	2.85 $\times \;10^{-12}$	12.26	0.001	11.67	25.48	35.16	56.84	692.10	91.14	69.78	1.477 $\times \;10^{-7}$
Road traffic	0.0121	3.353	0	5.14 $\times \;10^{-10}$	39.84	0	3.00 $\times \;10^{-8}$	3.40 $\times \;10^{-8}$	50.19	60.22	301.13	488.81	0	0
Sea traffic	0.0941	0	0	0.0626	0	0	0	0	75.17	300.69	500.76	109.87	0	0
Biogenic	0	0	5.023	0.0968	29.056	0	0	0.2975	0	20.094	854.02	0	0	76.83
Metal	0.2215	22.95	0	0	0	0	0	0	0	50.00	0	0	0	0

**Table A2:** Matrix $\Omega^{E}$ used in the simulations.

$\Omega^{E}$	Al	Cr	Fe	Mn	P	Sr	Ti	Zn	V	Ni	Co	Cu	Cd	Sb
Sea	0	1	1	1	0	0	1	1	1	1	1	1	1	1
Aged sea	1	0	1	1	0	0	0	1	1	1	0	0	1	1
Crustal	0	0	0	0	0	0	0	0	0	1	0	0	1	1
nitrates	0	0	0	0	0	1	1	0	1	1	0	0	0	0
sulfate	1	0	1	0	0	1	1	0	1	1	0	0	1	1
Biomass	0	1	0	0	1	0	1	0	1	1	1	1	1	0
Road traffic	1	1	0	0	0	1	1	0	1	1	0	0	1	0
Sea traffic	0	0	0	1	1	0	0	1	0	0	0	0	0	1
Biogenic	1	1	1	1	0	0	0	1	1	1	1	1	1	0
Metal	0	0	0	0	0	0	1	1	1	0	0	0	0	1
	**La**	**Pb**	**Na** ${}^{+}$	**NH** ${}_{4}^{+}$	**K** ${}^{+}$	**Mg** ${}^{2+}$	**Ca** ${}^{2+}$	**Cl** ${}^{-}$	**NO** ${}_{3}^{-}$	**SO** ${}_{4}^{2-}$	**OC**	**EC**	**Levo.**	**Polyols**
Sea	1	1	0	1	0	0	0	0	1	0	1	0	1	1
Aged sea	1	0	0	1	0	0	0	0	0	0	0	1	1	1
Crustal	0	0	0	0	0	0	0	1	0	0	0	1	1	1
nitrates	0	0	1	0	0	0	1	0	0	1	0	0	1	0
sulfate	1	0	1	0	0	0	1	0	1	0	0	0	1	1
Biomass	1	0	0	0	0	0	0	0	0	0	0	0	0	0
Road traffic	0	0	1	0	0	1	0	0	0	0	0	0	1	1
Sea traffic	0	0	1	0	1	0	1	0	0	0	0	0	1	1
Biogenic	1	1	0	0	0	0	1	0	0	0	0	1	1	0
Metal	0	0	1	0	1	0	1	1	1	0	1	1	1	1

**Table A3:** Matrix $F_{init}$ used in the simulations.

$F_{\mathrm{init}}$	Al	Cr	Fe	Mn	P	Sr	Ti	Zn	V	Ni	Co	Cu	Cd	Sb
Sea	0.2	1.00 $\times \;10^{-11}$	1.00 $\times \;10^{-11}$	1.00 $\times \;10^{-11}$	0.01	0.8	1.00 $\times \;10^{-11}$	1.00 $\times \;10^{-11}$	1.00 $\times \;10^{-11}$	1.00 $\times \;10^{-11}$	1.00 $\times \;10^{-11}$	1.00 $\times \;10^{-11}$	1.00 $\times \;10^{-11}$	1.00 $\times \;10^{-11}$
Aged sea	1.00 $\times \;10^{-11}$	0.001	1.00 $\times \;10^{-11}$	1.00 $\times \;10^{-11}$	1	1	0.01	1.00 $\times \;10^{-11}$	1.00 $\times \;10^{-11}$	1.00 $\times \;10^{-11}$	0.01	0.01	1.00 $\times \;10^{-11}$	1.00 $\times \;10^{-11}$
Crustal	200	0.001	150	2	2	2	20	2	2	1.00 $\times \;10^{-11}$	0.001	0.0001	1.00 $\times \;10^{-11}$	1.00 $\times \;10^{-11}$
nitrates	1.00 $\times \;10^{-5}$	2.00 $\times \;10^{-6}$	8	1	0.4	1.00 $\times \;10^{-11}$	1.00 $\times \;10^{-11}$	4	1.00 $\times \;10^{-11}$	1.00 $\times \;10^{-11}$	0.001	0.5	0.01	0.2
sulfate	1.00 $\times \;10^{-11}$	1.00 $\times \;10^{-4}$	1.00 $\times \;10^{-11}$	1.00 $\times \;10^{-4}$	0.5	1.00 $\times \;10^{-11}$	1.00 $\times \;10^{-11}$	0.4	1.00 $\times \;10^{-11}$	1.00 $\times \;10^{-11}$	0.01	1.00 $\times \;10^{-4}$	1.00 $\times \;10^{-11}$	1.00 $\times \;10^{-11}$
Biomass	5	1.00 $\times \;10^{-11}$	10	2	9.43 $\times \;10^{-11}$	0.001	1.00 $\times \;10^{-11}$	1	1.00 $\times \;10^{-11}$	1.00 $\times \;10^{-11}$	1.00 $\times \;10^{-11}$	1.00 $\times \;10^{-11}$	1.00 $\times \;10^{-11}$	1.006 $\times \;10^{-10}$
Road traffic	1.00 $\times \;10^{-11}$	1.00 $\times \;10^{-11}$	50	1	1.00 $\times \;10^{+0}$	1.00 $\times \;10^{-11}$	1.00 $\times \;10^{-11}$	24	1.00 $\times \;10^{-11}$	1.00 $\times \;10^{-11}$	1.00 $\times \;10^{-11}$	4	1.00 $\times \;10^{-11}$	2
Sea traffic	0.01	1.00 $\times \;10^{-4}$	0.4	1.00 $\times \;10^{-11}$	1.00 $\times \;10^{-11}$	0.1	1.00 $\times \;10^{-4}$	1.00 $\times \;10^{-11}$	18	10	1	1.00 $\times \;10^{-3}$	1.00 $\times \;10^{-4}$	1.00 $\times \;10^{-11}$
Biogenic	1.00 $\times \;10^{-11}$	1.00 $\times \;10^{-11}$	1.00 $\times \;10^{-11}$	1.00 $\times \;10^{-11}$	5	7.96 $\times \;10^{-10}$	7.96 $\times \;10^{-10}$	1.00 $\times \;10^{-11}$	1.00 $\times \;10^{-11}$	1.00 $\times \;10^{-11}$	1.00 $\times \;10^{-11}$	1.00 $\times \;10^{-11}$	1.00 $\times \;10^{-11}$	7.96 $\times \;10^{-10}$
Metal	73	70	650	50	3	5	1.00 $\times \;10^{-11}$	1.00 $\times \;10^{-11}$	1.00 $\times \;10^{-11}$	30	1	3	4	1.00 $\times \;10^{-11}$
	**La**	**Pb**	**Na** ${}^{+}$	**NH** ${}_{4}^{+}$	**K** ${}^{+}$	**Mg** ${}^{2+}$	**Ca** ${}^{2+}$	**Cl** ${}^{-}$	**NO** ${}_{3}^{-}$	**SO** ${}_{4}^{2-}$	**OC**	**EC**	**Levo.**	**Polyols**
Sea	1.00 $\times \;10^{-11}$	1 $\times \;10^{-9}$	320	5 $\times \;10^{-5}$	10	38	11	550	1 $\times \;10^{-5}$	70	1 $\times \;10^{-5}$	1 $\times \;10^{-5}$	9.98 $\times \;10^{-11}$	9.98 $\times \;10^{-11}$
Aged sea	1.00 $\times \;10^{-11}$	0.01	250	1 $\times \;10^{-8}$	1	40	15	150	320DA80.eps	210	12	1.00 $\times \;10^{-11}$	9.99 $\times \;10^{-11}$	9.99 $\times \;10^{-11}$
Crustal	0.0001	1 $\times \;10^{-7}$	0.0001	0.0001	10	10	250	1.00 $\times \;10^{-11}$	30	30	290	1.00 $\times \;10^{-11}$	1.00 $\times \;10^{-10}$	1.00 $\times \;10^{-10}$
nitrates	0.2	0.5	1 $\times \;10^{-10}$	300	5	1.00 $\times \;10^{-11}$	1.00 $\times \;10^{-11}$	0.2	600	1.00 $\times \;10^{-11}$	80	1.00 $\times \;10^{-11}$	1.00 $\times \;10^{-10}$	1.00 $\times \;10^{-10}$
sulfate	1.00 $\times \;10^{-11}$	0.1	1.00 $\times \;10^{-8}$	305	10	1.00 $\times \;10^{-11}$	1.00 $\times \;10^{-11}$	1.00 $\times \;10^{-3}$	1.00 $\times \;10^{-11}$	584	100	1.00 $\times \;10^{-11}$	1.00 $\times \;10^{-10}$	1 $\times \;10^{-11}$
Biomass	1.00 $\times \;10^{-11}$	1	3	28	72	5	38	66	66	66	510	70	57	9.43 $\times \;10^{-11}$
Road traffic	1	9.99	1.00 $\times \;10^{-10}$	1.00 $\times \;10^{-8}$	57	0.00049	1.00 $\times \;10^{-6}$	1.00 $\times \;10^{-11}$	79.99	80	260	430	9.99 $\times \;10^{-11}$	9.99 $\times \;10^{-11}$
Sea traffic	0.5	1.00 $\times \;10^{-8}$	1 $\times \;10^{-11}$	1.00 $\times \;10^{-2}$	1 $\times \;10^{-11}$	1 $\times \;10^{-11}$	1.00 $\times \;10^{-11}$	1 $\times \;10^{-11}$	110	250	450	160	8.37 $\times \;10^{-11}$	8.37 $\times \;10^{-11}$
Biogenic	1.00 $\times \;10^{-11}$	7.96 $\times \;10^{-10}$	1	1	9	4	1.00 $\times \;10^{-11}$	7.96 $\times \;10^{-10}$	5	5	800	1 $\times \;10^{-11}$	7.96 $\times \;10^{-10}$	170
Metal	1	40	1.00 $\times \;10^{-11}$	1.00 $\times \;10^{-2}$	1.00 $\times \;10^{-11}$	1.00 $\times \;10^{-2}$	1.00 $\times \;10^{-11}$	0.005	0.001	70	0.00164	1.64 $\times \;10^{-10}$	1.64 $\times \;10^{-10}$	1.64 $\times \;10^{-10}$

## Appendix C. Real Data Operating Conditions

When considering the real dataset, let us emphasize that we do not know in advance the profile matrix. The chemists are able to provide an initial profile matrix which is given in Table A4. As in the previous appendix, the same initial matrix is applied to both the informed and non-informed methods. ϵ is a very small quantity to make the initialization very close to the case of informed methods where set values are zeros.

**Table A4:** Matrix $F_{init}$ used in the real data case.

$F_{\mathrm{init}}$	Al	Cr	Fe	Mn	P	Sr	Ti	Zn	V	Ni	Co	Cu	Cd	Sb
Sea	0.19	ϵ	ϵ	ϵ	1.00	8.00	ϵ	ϵ	ϵ	ϵ	ϵ	ϵ	ϵ	ϵ
Aged sea	0.10	0.01	0.50	0.01	1.00	8.00	0.02	0.02	ϵ	ϵ	1.00	0.01	0.01	0.01
Crustal	266.67	0.14	150	2.00	ϵ	2.00	20	0.50	0.50	0.07	0.07	0.07	ϵ	0.01
nitrates	0.98	0.98	30	0.98	ϵ	0.98	0.98	20	ϵ	0.98	0.98	10	0.98	0.98
sulfate	1.00	1.00	30	1.00	ϵ	1.00	15.00	20	ϵ	1.00	1.00	1.00	1.00	1.00
Biomass	4.00	ϵ	9.00	1.00	ϵ	1.00	1.00	10	ϵ	ϵ	ϵ	1.00	ϵ	ϵ
Road traffic	20	1.00	50	5.00	ϵ	1.00	ϵ	50	5.00	10	5.00	50	5.00	50
Sea traffic	10	ϵ	10	ϵ	ϵ	ϵ	ϵ	5.00	55.00	55.00	30	ϵ	ϵ	ϵ
Biogenic	0.01	ϵ	0.01	ϵ	20	ϵ	ϵ	ϵ	ϵ	ϵ	ϵ	1.00	ϵ	ϵ
Metal	80	80	358	40	8	18.00	40	40	30	30	1.00	40	50	30
	**La**	**Pb**	**Na** ${}^{+}$	**NH** ${}_{4}^{+}$	**K** ${}^{+}$	**Mg** ${}^{2+}$	**Ca** ${}^{2+}$	**Cl** ${}^{-}$	**NO** ${}_{3}^{-}$	**SO** ${}_{4}^{2-}$	**OC**	**EC**	**Levo.**	**Polyols**
Sea	ϵ	ϵ	320.00	ϵ	10.00	40.00	10.00	540.08	ϵ	70.00	0.64	0.09	ϵ	ϵ
Aged Sea	ϵ	0.01	250.00	ϵ	10.00	25.00	10.00	200.00	275.30	210.00	8.00	1.00	ϵ	ϵ
Crustal	ϵ	0.14	10.00	3.00	100.00	70.14	210.00	7.00	20.00	35.07	90.00	12.62	ϵ	ϵ
nitrates	ϵ	0.98	ϵ	200.00	0.98	0.98	40.00	0.98	547.30	ϵ	100.00	40.00	ϵ	ϵ
sulfate	ϵ	20.00	ϵ	200.00	34.00	1.00	40.00	1.00	ϵ	554.00	60.00	16.00	ϵ	ϵ
Biomass	0.00	0.94	2.83	28.31	70.00	4.72	37.74	66.05	70.00	66.05	500.61	69.29	56.46	ϵ
Road traffic	ϵ	10.00	ϵ	10.00	10.00	ϵ	21.00	2.00	80.00	40.00	271.73	303.27	ϵ	ϵ
Sea traffic	15.00	10.00	ϵ	10.00	ϵ	ϵ	20.00	ϵ	10.00	30.00	580.00	160.00	ϵ	ϵ
Biogenic	ϵ	ϵ	1.00	1.00	5.00	4.00	1.00	ϵ	5.00	5.00	760.00	50.00	ϵ	146.98
Metal	1.00	80.00	ϵ	1.00	48.00	10.00	5.00	ϵ	ϵ	10.00	ϵ	ϵ	ϵ	ϵ

Prior set value information is provided through the specification of both $\Omega^{E}$ and $\Phi^{E}$. The set value configuration is the same as those presented in [54]. As a consequence, $\Phi^{E}$ is equal to $\Phi^{E}=0_{p\times m}$. The position of the set entries is provided in Table A5.

**Table A5:** Matrix $\Omega^{E}$ used in the real data case.

$\Omega^{E}$	Al	Cr	Fe	Mn	P	Sr	Ti	Zn	V	Ni	Co	Cu	Cd	Sb
Sea	0	1	0	1	0	0	1	1	1	1	1	1	1	1
Aged sea	0	0	0	0	0	0	0	0	1	1	0	0	0	0
Crustal	0	0	0	0	0	0	0	0	0	0	0	0	0	0
nitrates	0	0	0	0	0	0	0	0	1	0	0	0	0	0
sulfate	0	0	0	0	0	0	0	0	1	0	0	0	0	0
Biomass	0	1	0	0	0	0	0	0	1	1	0	0	0	0
Road traffic	0	0	0	0	0	0	0	0	0	0	0	0	0	0
Sea traffic	0	0	0	0	0	0	0	0	0	0	0	0	0	0
Biogenic	0	0	0	1	0	0	0	0	1	1	0	0	1	0
Metal	0	0	0	0	0	0	0	0	0	0	0	0	0	0
	**La**	**Pb**	**Na** ${}^{+}$	**NH** ${}_{4}^{+}$	**K** ${}^{+}$	**Mg** ${}^{2+}$	**Ca** ${}^{2+}$	**Cl** ${}^{-}$	**NO** ${}_{3}^{-}$	**SO** ${}_{4}^{2-}$	**OC**	**EC**	**Levo.**	**Polyols**
Sea	1	1	0	1	0	0	0	0	1	0	0	0	1	1
Aged sea	0	0	0	1	0	0	0	0	0	0	0	0	1	1
Crustal	0	0	0	0	0	0	0	0	0	0	0	0	1	1
nitrates	0	0	1	0	0	0	0	0	0	1	0	0	1	0
sulfate	0	0	1	0	0	0	0	0	1	0	0	0	1	0
Biomass	0	0	0	0	0	0	0	0	0	0	0	0	0	0
Road traffic	0	0	1	0	0	1	0	0	0	0	0	0	1	1
Sea Traffic	0	0	1	0	0	0	0	0	0	0	0	0	1	1
Biogenic	1	1	0	0	0	0	0	0	0	0	0	0	1	0
Metal	0	0	1	0	0	0	0	1	1	0	1	1	1	1

Prior bound information is provided through the specification of both $\Omega^{I}$ and $\Phi^{I+}$, $\Phi^{I-}$. For sake of concision, Table A6 only gathers the bound information.

**Table A6:** Matrices $\Phi^{I+}/\Phi^{I-}$ used in the real data case.

$\Phi^{I+}/\Phi^{I-}$	Al	Cr	Fe	Mn	P	Sr	Ti	Zn	V	Ni	Co	Cu	Cd	Sb
Sea	0	0	0	0	0	20/0	0	0	0	0	0	0	0	0
Aged sea	0	0	0	0	0	20/0	0	0	0	0	0	0	0	0
Crustal	400/50	0	200/1	0	0	0	40/0.001	0	0	0	0	0	0	0
nitrates	0	0	0	0	0	0	0	0	0	0	0	0	0	0
sulfates	0	0	0	0	0	0	0	0	0	0	0	0	0	0
Biomass	100/0.001	0	100/0.001	0	0	0	0	0	0	0	0	0	0	0
Road traffic	0	0	75/1	0	0	0	0	50/0.1	0	0	0	15/0.000001	0	15/0.000001
Sea traffic	0	0	70/0.1	0	0	0	0	0	70/5	70/5	50/0.00001	0	0	0
Biogenic	0	0	0	0	0	0	0	0	0	0	0	0	0	0
Metal	0	0	0	0	0	0	0	0	0	0	0	0	0	0
	**La**	**Pb**	**Na** ${}^{+}$	**NH** ${}_{4}^{+}$	**K** ${}^{+}$	**Mg** ${}^{2+}$	**Ca** ${}^{2+}$	**Cl** ${}^{-}$	**NO** ${}_{3}^{-}$	**SO** ${}_{4}^{2-}$	**OC**	**EC**	**Levo.**	**Polyols**
Sea	0	0	400/200	0	50/5	50/15	50/5	720/360	0	100/30	0	0	0	0
Aged sea	0	0	0	0	0	0	0	250/0	500/50	500/50	0	0	0	0
Crustal	0	0	0	0	150/5	150/5	500/50	0	50/0	40/0	0	0	0	0
nitrates	0	0	0	800/50	0	0	0	0	950/200	0	0	0	0	0
sulfates	0	0	0	800/50	0	0	0	0	0	950/200	0	0	0	0
Biomass	0	0	10/0	40/0	100/1	5/0	100/0.001	100/0.001	150/1	150/0	750/100	200/5	0	0
Road traffic	0	0	0	20/0	0	0	0	10/0	60/10	80/20	300/150	800/250	0	0
Sea Traffic	30/0	0	0	20/0	0	0	0	20/0	75/0	300/10	700/100	200/50	0	0
Biogenic	0	0	5/0	5/0	0	0	0	5/0	5/0	20/0	850/500	0	0	0
Metal	0	0	0	0	0	0	0	0	0	60/10	0	0	0	0
